# The calcium-binding protein CSE links Ca^2+^ signaling with cell-cell communication in cyanobacteria

**DOI:** 10.1038/s44318-026-00893-y

**Published:** 2026-08-13

**Authors:** Teresa A Müller, Felicia Stahlecker, Karolina Roganowicz, Sherihan Samir, Gregor L Weiss, Murray Coles, Khaled A Selim

**Affiliations:** 1 Interfaculty Institute of Microbiology and Infection Medicine, Cluster of Excellence “Controlling Microbes to Fight Infections - CMFI”, Tübingen University, Tübingen, Germany; 2 Department of Protein Evolution, Max Planck Institute for Biology, Tübingen, Germany; 3 Institute of Molecular Biology and Biophysics, Department of Biology, ETH Zürich, Zürich, Switzerland; 4 Institute of Medical Microbiology, University of Zürich, Zürich, Switzerland; 5 Microbial Biochemistry Group, Institute of Phototrophic Microbiology, CEPLAS Cluster of Excellence and Interfaculty Center for Membrane Research – iCMR, Heinrich-Heine University Düsseldorf, Universitätsstraße 1, 40225 Düsseldorf, Germany

**Keywords:** Cell Adhesion, Polarity & Cytoskeleton, Signal Transduction, Structural Biology

## Abstract

Multicellular cyanobacteria have evolved sophisticated cell–cell communication machinery to exchange, synchronize, and coordinate the efforts of individual cells. Analogous to gap junctions that have traditionally been regarded as a eukaryotic feature, multicellular cyanobacteria coordinate their cell–cell communication via septal junctions (SJs). However, the signals that regulate cell–cell communication and septal-junction assembly are largely unknown. Lately, calcium signaling has been implicated in regulating cell junctions in eukaryotes. We recently discovered a Ca^2+^-binding protein, CSE, which is exclusively found in multicellular cyanobacteria. Here, we investigate CSE as a potential link between calcium signaling and cell–cell communication. We solve the NMR structure of CSE in its Ca^2+^-bound state and revealed that CSE acts as Ca^2+^-buffer protein. Using cryo-electron tomography, we find that Δ*cse* mutant cells display significantly fewer septal junctions as well as SJ precursors known as nanopores. This indicates that CSE is not only essential for Ca^2+^ homeostasis, but also mediates cell–cell communication via regulating SJs and nanopores formation, and establishes Ca^2+^ signaling and CSE as key players regulating cyanobacterial multicellularity. Furthermore, these findings highlight calcium signaling as a conserved principle for regulating cellular junctions in organisms that diverged a billion years ago.

## Synopsis

Multicellular cyanobacteria like *Nostoc* sp. PCC 7120 build long filaments consisting of connected neighboring cells that exchange molecules through so-called septal junctions. This study shows that loss of the small calcium-binding protein CSE causes disruption of septal junctions and filament fragmentation.

- The NMR structure of CSE in its Ca^2+^-bound state reveals Ca^2+^-induced folding.
- The absence of CSE causes a strong fragmentation phenotype down to small filaments.
- In ∆*cse* mutant, the septal disks connecting neighboring cells have an increased area with a reduced number of septal junctions and their nanopore precursors.
- Cells lacking CSE show decreased cell-cell communication speed.

## Abstract (web summary)

Loss of CSE in cyanobacterium *Nostoc* sp. PCC 7120 reveals calcium signaling as an evolutionarily conserved regulator of multicellularity and cell-cell junctions.

## Graphical abstract

**Figure:**
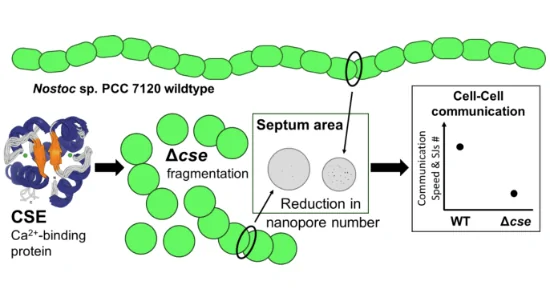

## Introduction

Calcium (Ca^2+^) ions have the distinction of being both essential and toxic to living cells. Their toxicity stems from the ability to bind and precipitate intracellular phosphate ions, as well as their negative effect on ribosome functions (Akanuma et al, 2014; Clapham, 2007; Weiss et al, 1973). Therefore, the absolute cellular Ca^2+^ concentrations must be held at nanomolar levels. To achieve this, free calcium ions are either excreted via channels or actively transported through pumps. Further, specific proteins bind calcium ions to buffer the free intracellular Ca^2+^ concentration. This tight control in turn leads to their essential role as a second messenger in a myriad of regulatory processes. Details of these systems have been elucidated over many years. This is particularly true for eukaryotes (Lehne et al, 2022), but some prokaryotic systems are now receiving more attention (Agaras et al, 2023; Burch-Konda et al, 2024; King et al, 2020; Kolodkin-Gal et al, 2023). An example is cyanobacteria, which share common ancestors with plant chloroplasts and have thus been used as models for studying photosynthesis. In plants, vital roles for Ca^2+^-signaling in regulating plant immunity and stress response have been recognized (Aratani et al, 2023; Hagihara et al, 2022; Toyota et al, 2018; Yan et al, 2024). In cyanobacteria, Ca^2+^-signaling is crucial for stress responses to reactive oxygen species (ROS) (Singh et al, 2016), changes in pH (Giraldez-Ruiz et al, 1997), temperature, salt, or osmotic stress (Mantovani et al, 2023; Torrecilla et al, 2000, 2001, 2004). Calcium ions are also implicated in controlling photosynthesis, regulating specific sugar and fatty acid metabolic pathways (Singh et al, 2016, 2017), and fine-tuning carbon/nitrogen metabolism by yet unknown mechanisms (Mantovani et al, 2023; Walter et al, 2016, 2019).

Filamentous cyanobacteria are emerging as model organisms for studying multicellularity, as they evolved sophisticated cell–cell communication machinery to exchange, synchronize, and coordinate the efforts of individual cells for the maximum benefit of the filament. By virtue of their multicellularity, some filamentous cyanobacteria have the ability to differentiate into specialized cells to fulfill specific metabolic tasks, e.g., heterocysts or akinetes (Flores and Herrero, 2010; Muro-Pastor and Maldener, 2019).

For instance, heterocysts are specialized cells that fix atmospheric nitrogen to ammonia using nitrogenases, the only known family of enzymes catalyzing this reaction under nitrogen-limiting conditions. For long, it has been known that calcium signaling is essential for proper heterocyst formation (Smith et al, 1987). A transient increase in Ca^2+^ concentration has been observed in pro-heterocysts shortly after nitrogen depletion (Torrecilla et al, 2004; Zhao et al, 2005). The role of Ca^2+^-binding proteins in this process has become more evident (Mantovani et al, 2023; Zhao et al, 2005; Walter et al, 2020). In the filamentous model organism *Nostoc* sp. PCC 7120 (hereafter *Nostoc*), two calcium-binding proteins have been implicated in heterocyst formation: CcbP (Cyanobacterial calcium binding Protein; Zhao et al, 2005) and CSE (Ca^2+^-Sensor EF-hand; Walter et al, 2019). CcbP is negatively regulated via the heterocyst transcription regulators NtcA and HetR (Forchhammer and Selim, 2020) and appears to be absent in heterocysts (Zhao et al, 2005; Shi et al, 2006). Recently, we identified CSE as a highly conserved protein in filamentous, nitrogen-fixing cyanobacteria and proved its importance for filament integrity, heterocyst differentiation, and photosynthesis (Walter et al, 2019, 2020). The overexpression of *cse* (*cse* OE) impaired both growth and photosynthesis (Walter et al, 2019). In contrast, *cse* deletion (Δ*cse*; encoded by *asr1131*) resulted in strong fragmentation of the filaments and negatively influenced heterocyst functions (Walter et al, 2020). Thus, both CSE and CcbP are involved in heterocyst differentiation, highlighting a conserved role for Ca^2+^-signaling in controlling cell differentiation; however, their specific functions and regulatory targets remain unknown.

The role of calcium-binding proteins in heterocyst formation hints at a wider role in cell–cell communication. In multicellular filamentous cyanobacteria, like *Nostoc*, the exchange of intracellular molecules along the filament is crucial both for heterocyst development and diazotrophic growth, as both heterocyst and vegetative cells rely on nutrients from each other (Flores and Herrero, 2010; Maldener et al, 2014). To exchange molecules between sister cells, these metabolites must cross two cytoplasmic membranes and a septal peptidoglycan (PG) disc within the septum between neighboring cells in the filament. Septal junctions (SJs) traverse this PG layer via nanopore arrays drilled by AmiC amidases (N-acetylmuramyl-L-alanin amidases) (Bornikoel et al, 2017; Lehner et al, 2013; Nürnberg et al, 2015), establishing a connection between the cytoplasm of neighboring cells (Lehner et al, 2013; Flores et al, 2016; Hoiczyk and Baumeister, 1995). SJs are multi-protein complexes, consisting of a septum-spanning tube with a membrane-embedded plug (likely formed by FraD/SepN proteins) at both ends, and a cap covering the plug on the cytoplasmic side (Kieninger et al, 2022; Weiss et al, 2019). The purified PG discs of *Nostoc* can harbor 80-150 nanopores per array. Mutants with a disruption in one of the AmiC genes or FraD have a highly reduced number of nanopores and are impaired in cell–cell communication (Bornikoel et al, 2017). Many of those mutants also suffer from strong fragmentation phenotypes and impaired heterocyst development, in analogy to the Δ*cse* phenotype (Walter et al, 2020; Kieninger, 2021; Kieninger and Maldener, 2021; Merino-Puerto et al, 2010; Merino-Puerto et al, 2011). Thus, there appears to be a strong connection between heterocyst development, filament integrity, and cell–cell communication. However, the molecular signals that control the cell–cell communication and the assembly of the SJs are absolutely unknown. Given that fluctuation of intracellular Ca^2+^ levels seems to influence cell–cell communication and filament integrity (Walter et al, 2020; Kieninger, 2021), we hypothesized that cyanobacterial cell–cell communication is potentially regulated by the Ca^2+^-binding protein CSE.

Here, we investigated the CSE protein as a potential link between intracellular Ca^2+^ and cell–cell communication. We solved the solution NMR structure of CSE in its Ca^2+^-bound state and revealed that CSE acts as Ca^2+^-buffer protein. Using cryo-electron tomography (cryo-ET), we show that CSE is not only essential for Ca^2+^-homeostasis, but also mediates the cell–cell communication via regulating the nanopores formation as the Δ*cse* mutant showed a strong variation in nanopore numbers coupled to a reduction in SJs. This determines, for the first time, Ca^2+^ as a novel factor controlling cell–cell communication and establishes CSE as a key player regulating cyanobacterial multicellularity.

## Results

### The correct folding of the CSE protein depends on calcium binding

CSE is a small protein of 77 amino acid residues that is monomeric when heterologously purified as a His_6_-tagged protein. Its sequence is highly conserved among N_2_-fixing multicellular cyanobacteria and contains two canonical, 12-residue EF-hand Ca^2+^-binding motifs (Appendix Fig. S1). The EF-hand motif is a characteristic Ca^2+^-binding domain that consists of a helix–loop–helix structural motif, in which the two α-helices (helices E and F) are connected by a loop coordinating Ca^2+^-binding (Kawasaki and Kretsinger, 1994). Accordingly, AlphaFold predicts the CSE structure with high confidence; the model shows two structurally similar Ca^2+^-binding sites, linked by an α-helix and contacting each other via a short β-bridge. It was therefore surprising that our crystallization attempts, in the presence or absence of Ca^2+^, to determine the mode of Ca^2+^-binding to CSE were unsuccessful.

**Figure EV1:**
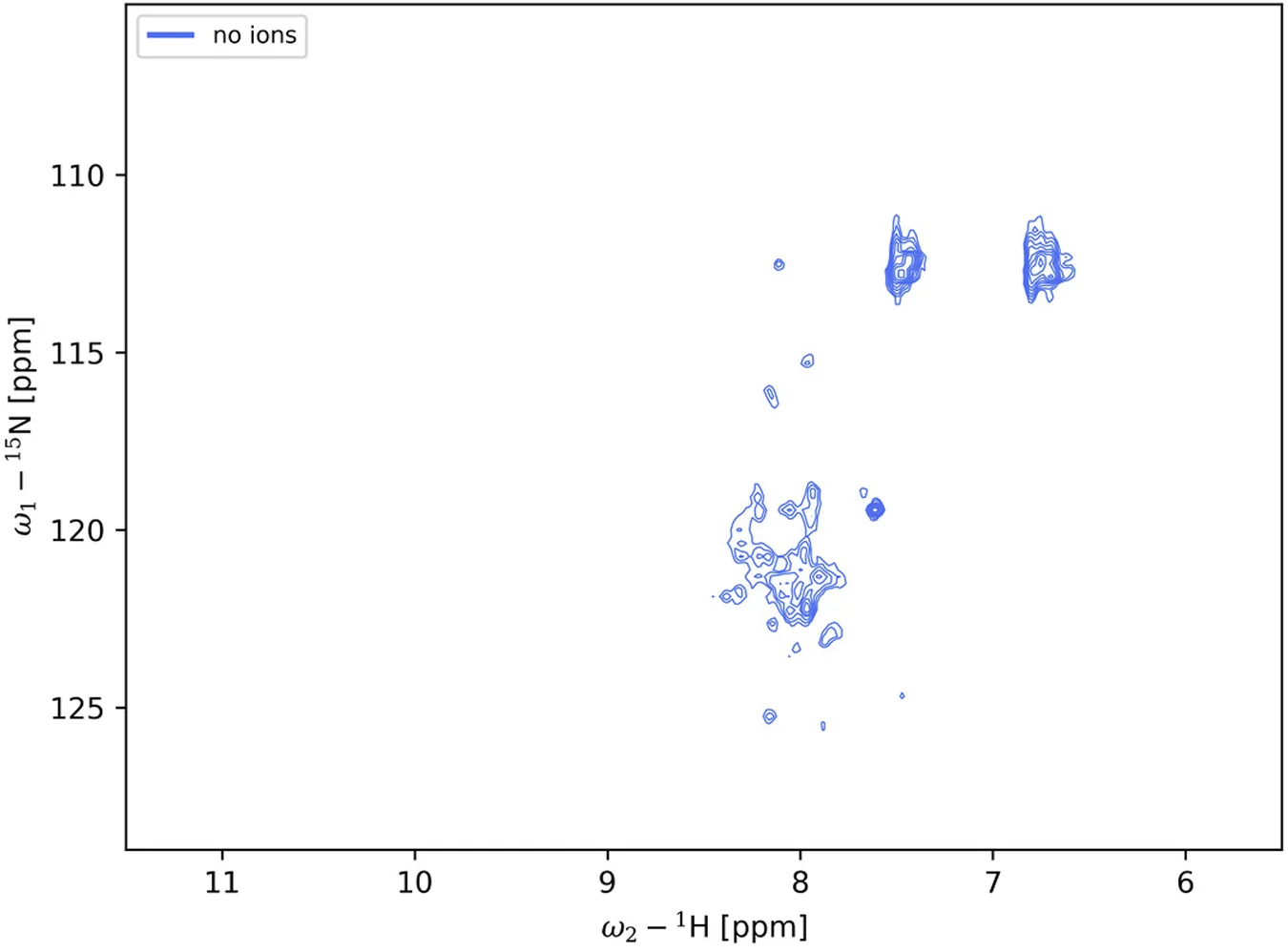
^15^N-HSQC spectrum of the ion-free CSE protein (i.e., without Mg^2+^ or Ca^2+^ ions) from *Nostoc* sp. PCC 7120.

Our previous results suggested that CSE undergoes substantial conformational changes upon Ca^2+^-binding (Walter et al, 2019). To explore this in more detail, we used NMR spectroscopy to determine the solution NMR structure of CSE and to investigate the dynamic of Ca^2+^-binding. For this purpose, we expressed ^13^C, ^15^N-labeled CSE and prepared an ion-free sample, using EDTA to sequester any divalent cations. The ^15^N-HSQC spectrum of this ion-free sample showed minimal peak dispersion, characteristic of an unfolded protein (Fig. EV1). Notably, two characteristic signals of folded EF-hand proteins, corresponding to glycine residues in position 6 of the EF-hand motif (Appendix Fig. S1), were absent from this spectrum (Fig. EV1). Similarly, the 1D ^1^H spectrum also displayed little peak dispersion, with only a few defined methyl proton peaks, indicating the existence of a minimal hydrophobic core. This suggested that CSE adopts a molten globule state in the absence of Ca^2+^, consistent with our previous circular dichroism (CD) measurements (Walter et al, 2019). We therefore conclude that CSE is substantially unfolded in an ion-depleted state.

**Figure EV2:**
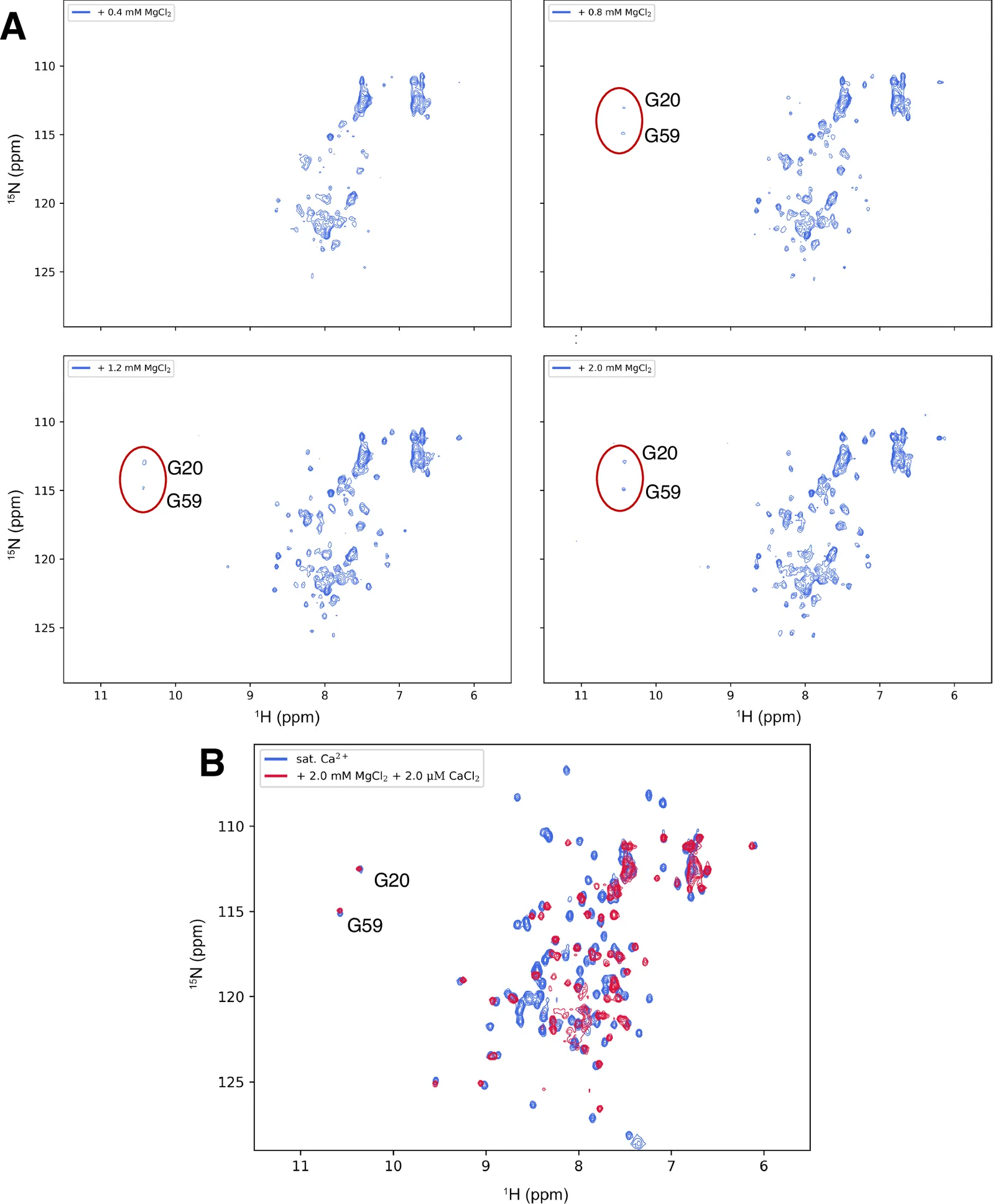
^15^N-HSQC spectra of CSE with Mg^2+^ and/or Ca^2+^. (**A**) ^15^N-HSQC spectra of CSE samples titrated with increasing concentrations of MgCl_2_ (0.4, 0.8, 1.2 and 2.0 mM, as indicated). Peaks corresponding to the characteristic glycine residues in position 6 of the EF-hand loops (G20 and G59) are labeled. (**B**) An overlay of the ^15^N-HSQC spectra of CSE saturated with Ca^2+^ (blue) and CSE titrated with 2 mM Mg^2+^ (red). The considerable differences in both peak positions and overall dispersion indicate that Ca^2+^ efficiently competes with Mg^2+^ for binding sites.

**Figure 1:**
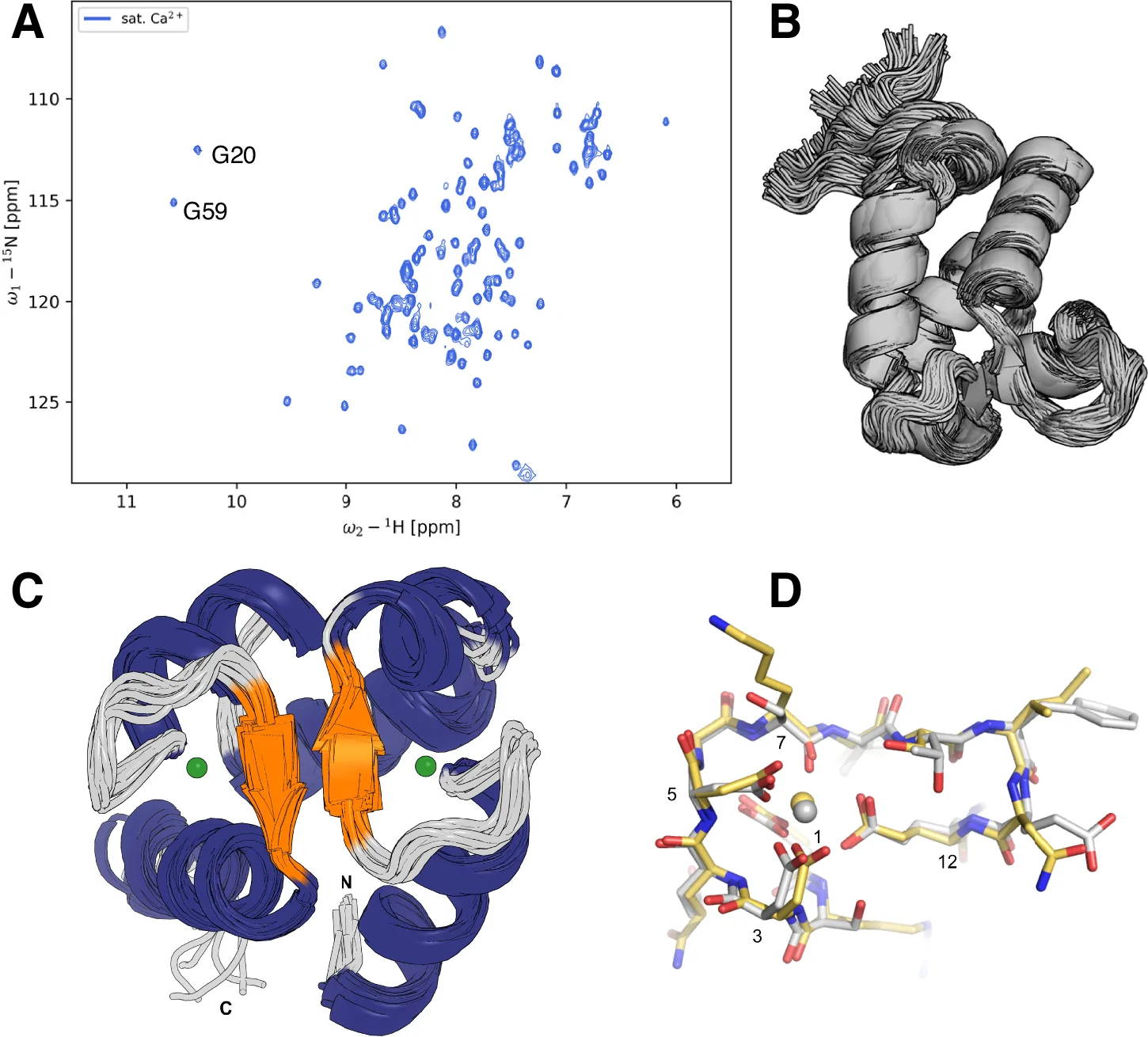
Structure of Ca^2+^-binding protein CSE. (**A**) The ^15^N-HSQC spectrum of Ca^2+^-saturated CSE protein shows good peak dispersion, indicating that the protein is well-folded. Two glycine residues in position 6 of the EF-hand loops (G20 and G59) are labeled. Their amide protons show an unusual downfield shift of 10–11 ppm, which is common among EF-hand proteins. (**B**) First 500 structural frames of solution NMR structure of CSE generated using CoMAND. (**C**) The final CoMAND ensemble for CSE colored by secondary structure. The positions of the N- and C-termini are marked. A bound Ca^2+^ ion is shown for the first model (green sphere). The backbone RMSD for superimposition of the models to the average structure is 0.66 Å. (**D**) Close-up of the CSE Ca^2+^-binding sites. The N-terminal (yellow) and C-terminal (gray) EF-hand loops are shown superimposed over backbone atoms (with RMSD 0.41 Å). Residues that coordinate the Ca^2+^ ion (yellow or gray sphere for N- and C-terminal sites, respectively) are numbered according to their positions in the 12-residue EF-hand motif (corresponding to D15, D17, D19, K21, and E26 at the N-terminal binding site; and D54, D56, D58, S60, and E65 at the C-terminal binding site; Appendix Fig. S1).

Our CD analysis indicated that both Ca^2+^ and Mg^2+^ ions can substantially induce structural changes upon binding to CSE, evident from characteristic α-helical spectra with two defined minima at 207 nm and at 222 nm (Walter et al, 2019). To further investigate the effect of divalent cations, we first titrated the ion-free CSE sample with Mg^2+^, the most abundant divalent metal ion in the cytoplasm. Increasing Mg^2+^ concentration gradually improved the peak dispersion of the ^15^N-HSQC spectra, approaching that of a well-folded protein (Fig. EV2). The two signals expected for the glycine residues at position 6 (Appendix Fig. S1) of the EF-hand loops were now clearly identified at 10-11 ppm (Fig. EV2A), serving as markers of a folded EF-hand. This indicates that Mg^2+^ considerably contributes to structuring the protein. However, even at 2 mM MgCl_2_, we were unable to record triple-resonance experiments for assignment and structure determination. This is presumably due to substantial conformational exchange associated with weaker Mg^2+^-binding, consistent with a high dissociation constant (K_D_) for Mg^2+^ (Walter et al, 2019). The addition of 2 µM CaCl_2_ to the same sample further enhanced peak dispersion in the ^15^N-HSQC spectrum, supporting the high specificity and affinity of Ca^2+^ binding to CSE. Notably, even at high Mg^2+^ concentration (2 mM), Ca^2+^ displaced Mg^2+^ from the binding sites. Although this sample allowed recording triple-resonance experiments, the quality of the spectra was insufficient for full sequential assignment, likely due to unfavorable exchange dynamics from competition of Mg^2+^ and Ca^2+^ for the EF-hand binding sites. Ultimately, we obtained high-quality spectra for structure determination only from a freshly prepared, ion-free sample saturated with Ca^2+^. In this Ca^2+^ saturated state, the ^15^N-HSCQ spectrum exhibited well-dispersed signals with characteristic EF-hand glycine fingerprints (Fig. 1A), consistent with a well-folded protein. Moreover, comparing the Ca^2+^- and Mg^2+^-saturated samples (Fig. EV2B) revealed that several peaks shifted or disappeared in the spectrum of the Mg^2+^-saturated sample, further supporting the specificity of Ca^2+^ binding for proper CSE folding.

To determine the solution NMR structure of Ca^2+^-saturated CSE, we applied the CoMAND structure determination protocol (ElGamacy et al, 2019), which we have developed to provide an ensemble that best explains the input data and therefore reflects native-like conformational diversity. We first performed preliminary calculations without modeling of bound calcium (Fig. 1B) to confirm that residues within the EF-hand loops were well defined and positioned to make the expected pentagonal bipyramidal contacts with the ion. The CoMAND method selects frames from equilibrium molecular dynamics to best explain input NMR data from NOESY spectra. In order for these simulations to produce a realistic distribution of charges, it was necessary to model calcium ions into both sites (see “Methods”). The final ensemble was selected from a pool of 2460 frames drawn from these simulations. The resulting ensemble is shown in (Fig. 1C). Structure statistics for the final ensemble are presented in (Appendix Table S1), and the final ensemble was deposited under the PDB accession code (9QSB).

The Ca^2+^-bound form of CSE adopts the typical structure of a dual EF-hand protein, with two helix–loop-helix units containing the EF-hand motifs (Fig. 1C) linked by a small β-sheet. This agrees closely with the AlphaFold model, with only minor differences in backbone conformations, where the final ensemble explains the data better than the starting model. The Ca^2+^-binding sites have canonical, EF-hand binding modes, with five chelating residues providing 6 ion contacts. In each case, these involve the side chains of aspartate residues in positions 1, 3, and 5 of the EF-loop, a backbone carboxyl of the residue in position 7, and a bidentate contact to the side chain of a glutamate residue in position 12 (Appendix Fig. S1). The seventh ion contact expected for pentagonal bipyramidal geometry is presumably provided by the solvent. In the N-terminal site, these are D15, D17, D19, K21, and E26, while at the C-terminus, these are D54, D56, D58, S60, and E65 (Fig. 1D). The two loops in these structures can be superimposed with an RMSD of 0.4 Å. The CoMAND method allows a detailed analysis of the conformational diversity of these residues. In all cases, the NOESY data is well explained by a single conformation, with the R-factor expressing the match between experimental and back-calculated data approaching that of noise.

**Figure 2:**
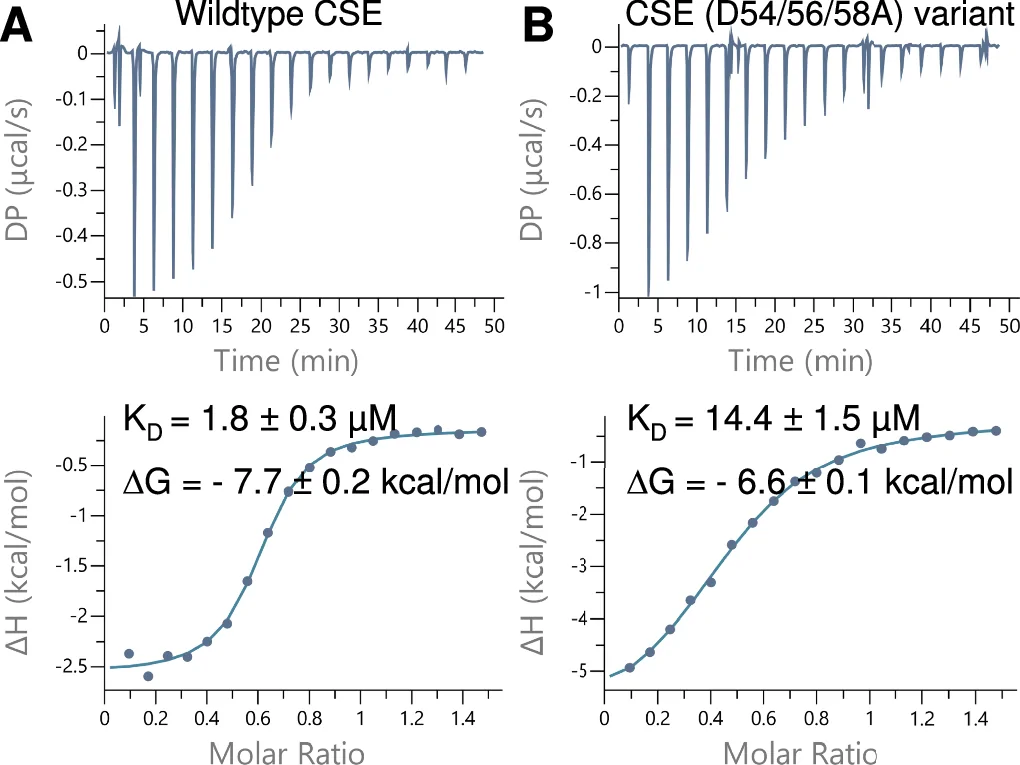
ITC analysis of Ca^2+^-binding to CSE proteins. (**A**) wild-type CSE protein and (**B**) CSE point mutation variant (D54/56/58A). Upper panels show the raw ITC data in the form of heat produced during the titration of 1 mM Ca^2+^ on 132 µM monomeric concentration of CSE proteins. Lower panels show the binding isotherms and the best-fit curves according to the one binding site model with K_D_ values and Gibbs free energy (∆G), as indicated.

To further elaborate on the importance of EF-loops for Ca^2+^-binding, we created a point mutant variant (D54/56/58 A), in which we replaced the D54, D56, and D58 of the C-terminal Ca^2+^-binding site by alanine(s). Next, we measured the Ca^2+^-binding affinity to this triple mutant variant compared to the wild-type CSE protein using isothermal titration calorimetry (ITC; Fig. 2A,B). The CSE (D54/56/58A) variant showed a reduced ability to bind Ca^2+^ with 8-fold decrease in the binding affinity (K_D_ of 14.4 ± 1.5 µM; Fig. 2B) compared to wild-type CSE protein (K_D_ of 1.8 ± 0.3 µM; Fig. 2A). Moreover, it showed a reduction in the Gibbs free energy (∆G) with −6.6 ± 0.1 kcal/mol compared to wild-type CSE of −7.7 ± 0.2 kcal/mol, indicating that Ca^2+^-binding happens more spontaneously for the wild-type CSE to reach the equilibrium. This result further supports the role of the EF-loops in mediating the Ca^2+^-binding specificity of CSE.

**Figure EV3:**
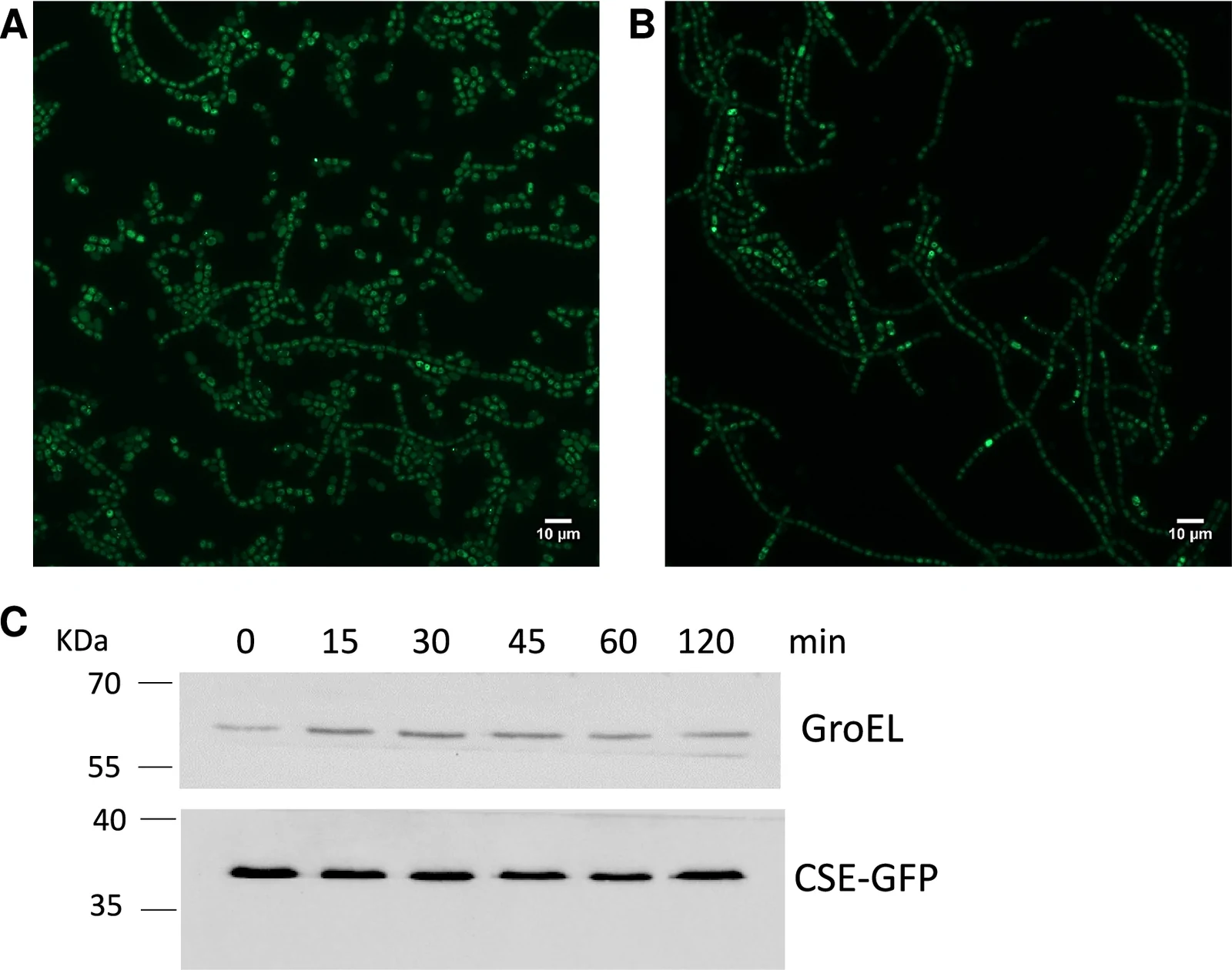
Cellular localization and abundance of CSE-sfGFP under the control of the native *cse* promoter. (**A**, **B**) CSE-sfGFP shows cytoplasmic localization, whether *cse-sfgfp* is integrated into the genome (**A**; pMBA226 strain) or expressed from the α-plasmid of *Nostoc* (**B**; pMBA224 strain). Scale bar: 10 µm. Both pMBA226 and pMBA224 are a kind gift from Manuel Brenes-Álvarez (Freiburg University). (**C**) Immunoblot analysis of CSE-sfGFP ( ≈ 37 kDa) expression using specific α-GFP antibodies under 1% CO_2_ and after shifting the cells to 2 h of darkness compared to the expression of GroEL (used as a control marker). The immunoblot analysis revealed that the CSE-sfGFP is abundant in the cell compared to GroEL.

Finally, to have an impression about the cellular abundance of CSE protein, a strain expressing CSE-sfGFP fusion was constructed, allowing to visualize the in vivo CSE expression and localization (Fig. EV3A,B). CSE-sfGFP appeared to be cytoplasmic (Fig. EV3A,B), and immunoblotting analysis using specific GFP antibodies revealed that CSE-GFP is relatively abundant compared to the GroEL marker (Fig. EV3C). Taken together, the NMR and the ITC binding data, along with the cellular abundance of CSE, support the idea that CSE acts as a Ca^2+^-buffer protein, similar to other EF-hand containing proteins involved in the control of Ca^2+^-homeostasis (Domínguez et al, 2015; Yonekawa et al, 2005; Zhao et al, 2010), while it becomes unfolded in the absence of divalent cations.

### CSE controls cell–cell communication in multicellular cyanobacteria

**Figure 3:**
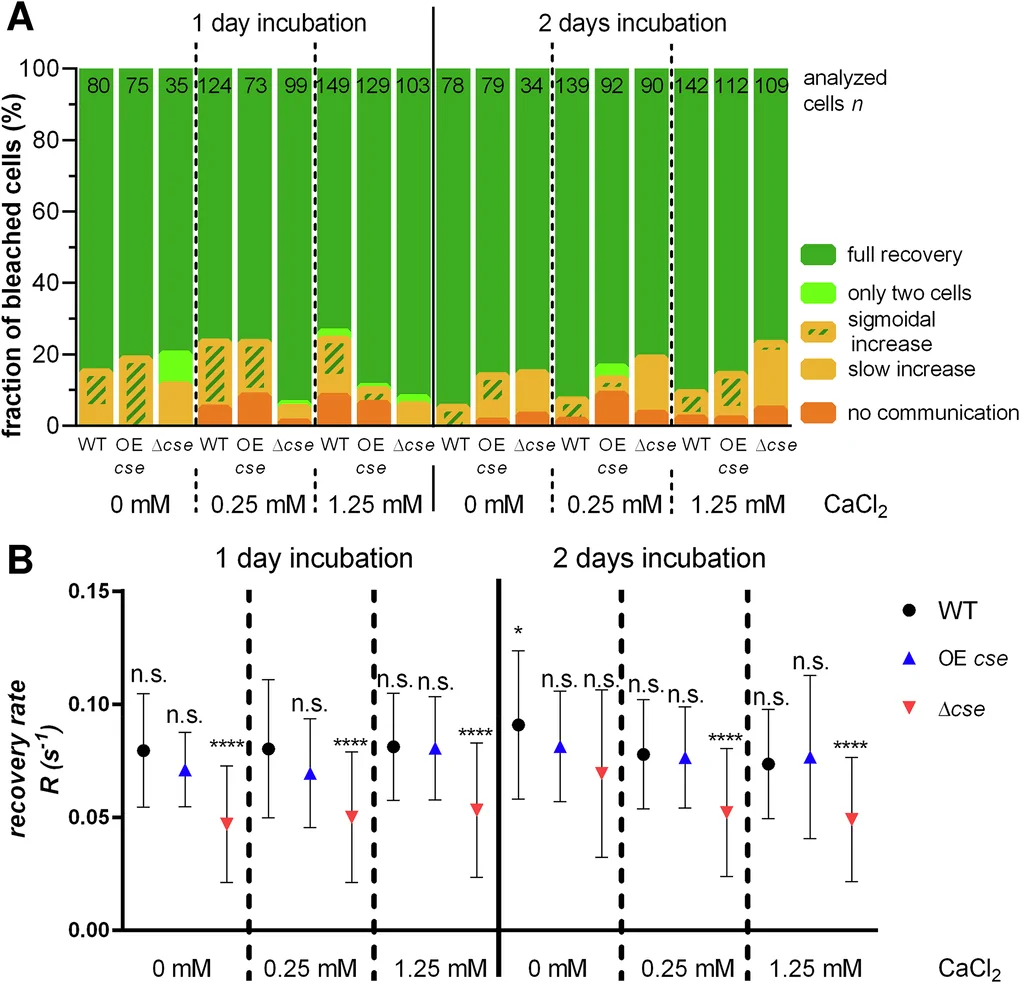
Cell–cell communication analysis via FRAP of *Nostoc* sp. PCC 7120 wild-type (WT) strain and its derived *cse* knockout (Δ*cse*) and overexpression (*cse* OE) mutants under different Ca^2+^ supplementation (0 mM, 0.25 mM, and 1.25 mM). (**A**) Cell–cell communication of normal-looking long filaments. The cells were incubated for one or two days with the indicated Ca^2+^ concentrations prior to the FRAP experiment and showing 5–15% of the cells exhibiting a sigmoidal increase (see an example in Fig. EV4A). Pooled data is shown from at least biological quadruplets, except for the Δ*cse* mutant at 0 mM CaCl_2_ (with biological duplicates). The number of cells analyzed is indicated. (**B**) Communication rates of *cse* mutants, calculated from FRAP analysis in (**A**) for fully communicating cells (in green), and showing very slow communication for the Δ*cse* mutant. The results represent the mean recovery rates with standard deviation of fully recovering cells (in green of **A**). Significance *P value = 0.039; ****P value < 0.0001 in one-way ANOVA analysis, in comparison to WT. n.s. not significant. Source data are available online for this figure.

**Figure EV4:**
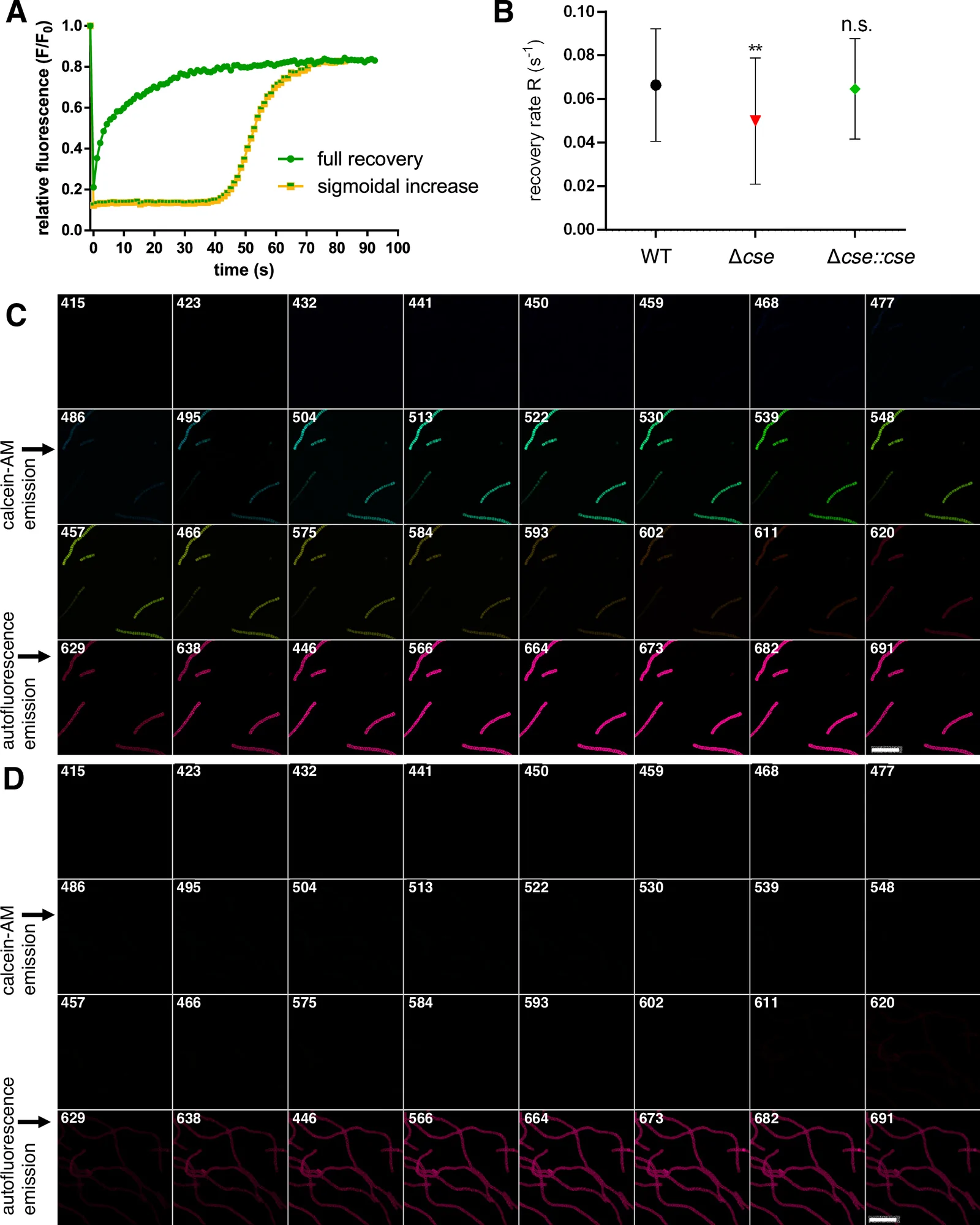
Cell–cell communication analysis via FRAP of wild-type (WT) *Nostoc* sp. and its derived *cse* knockout (Δ*cse*) and complementation (Δ*cse::cse*) strains. (**A**) An example of a sigmoidal recovery curve (in yellow) compared to full recovery response (in green) over time. The sigmoidal increase is characterized by a delay of up to 40–50 s of the fluorescence signal, then rapidly increases, like a full recovery response. First time point is before bleaching (at 1.0 F/F_0_) and time point *t* = 0 is measured directly after bleaching (at 0.1–0.2 F/F_0_). (**B**) Communication rates calculated from fully recovering cells from FRAP analysis, and showing that the Δ*cse::cse* complementation strain exhibits wild-type (WT) like communication, while Δ*cse* shows a slow communication rate. Significance **P value = 0.006 for Δ*cse* and *P* = 0.73 (not significant) for Δ*cse::cse* complementation strain in Student’s *t* test, in comparison to WT. n.s. not significant. Data are pooled from three independent biological replicates with standard deviation (WT, *n* = 298 cells; Δ*cse::cse*, *n* = 287 cells). (**C**, **D**) Lambda scan analysis of *Nostoc* cells excited at 405 nm after staining with (**C**) or without calcein-AM (**D**). Scale bar: 20 µm. Confocal fluorescence micrographs for emission measured in a range from 415 nm to 691 nm (step size of 8.625 nm). Lambda scan analysis revealed no overlap between calcein-AM emission (**C**) and cyanobacterial autofluorescence (**D**). Source data are available online for this figure.

Given that Ca^2+^ seems to influence cell–cell communication (Kieninger, 2021) and the *cse* deletion (Δ*cse*) mutant induces a strong fragmentation phenotype (Walter et al, 2020), we decided to systematically investigate the ability of CSE to control cell–cell communication in *Nostoc*. For this purpose, we used FRAP (fluorescence recovery after photobleaching) and compared the Δ*cse* knockout and *cse* overexpression (*cse* OE) mutants to wild-type cells of *Nostoc*. To test if calcium concentrations influence cell–cell communication properties, all strains were cultivated for one or two days in BG11 medium with either 0 mM, 0.25 mM (standard BG11 medium), or 1.25 mM CaCl_2_. Under all tested conditions, most of the cells (75–90%) of all strains showed a qualitative full recovery of the fluorescence signal and, thereby, a normal cell–cell communication behavior (Fig. 3A). Also, about 5–15% of the cells exhibited a sigmoidal and/or slow increase in the fluorescence recovery, which was barely recognized for Δ*cse* cells (Fig. 3A). The sigmoidal increase is characterized by a delay of up to 40–50 s of the fluorescence signal, then rapidly increases, like a full recovery response (Fig. EV4A). Notably, this type of sigmoidal fluorescence recovery has not been previously reported in cyanobacteria FRAP studies, for which the reasons are not yet clear. Furthermore, neither the extracellular Ca^2+^ concentrations nor the incubation time (1 and 2 days) impacts the cell–cell communication for any strain (Fig. 3A).

For qualitative analysis, we measured the recovery rate of the fluorescent signal (Fig. 3B; Appendix Table S2) for only the communicating cells (in green of Fig. 3A). Under all tested conditions, the wild-type cells showed a mean recovery rate of 0.08 ± 0.027 per second (Fig. 3B), which was comparable to the previously measured wild-type cells 0.09 ± 0.020 (Weiss et al, 2019) (see the raw data; Appendix Table S2). The *cse* OE strain exhibited a similar rate (0.076 ± 0.025 per second) to the wild-type cells, while the Δ*cse* mutant communicated significantly slower (0.052 ± 0.029 per second; Fig. 3B), implying a potential change in the SJs machinery (i.e., SJs are not fully operative). To test whether the slow communication phenotype depends mainly on the absence of CSE, we complemented the Δ*cse* mutant with the *cse* gene under its native promoter using the low-copy number plasmid *pRL1342* (Walter et al, 2019, 2020). The complemented Δ*cse*::*cse* strain showed a normal communication rate comparable to the WT cells (Fig. EV4B), supporting a role for CSE in regulating cyanobacterial cell–cell communication, likely through controlling Ca^2+^-homeostasis as a Ca^2+^-buffer protein.

**Figure 4:**
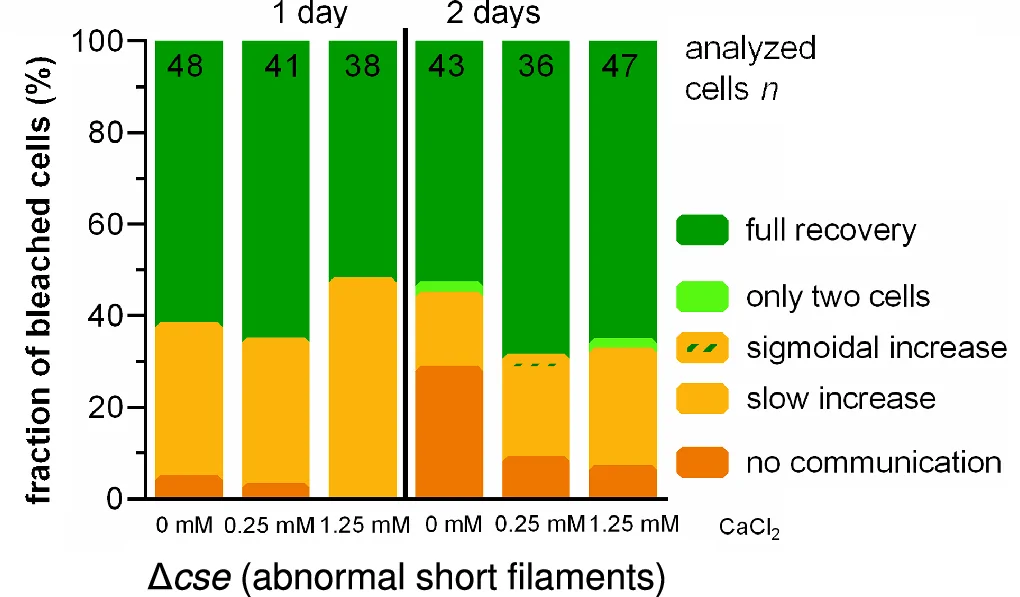
Cell–cell communication analysis via FRAP. FRAP analysis of the heavily fragmented short filaments of the Δ*cse* mutant (under different Ca^2+^ supplementation: 0 mM, 0.25 mM, 1.25 mM, as indicated), showing strong impairment of cell–cell communication with even 10–30% completely non-communicating cells. Source data are available online for this figure.

**Figure 5:**
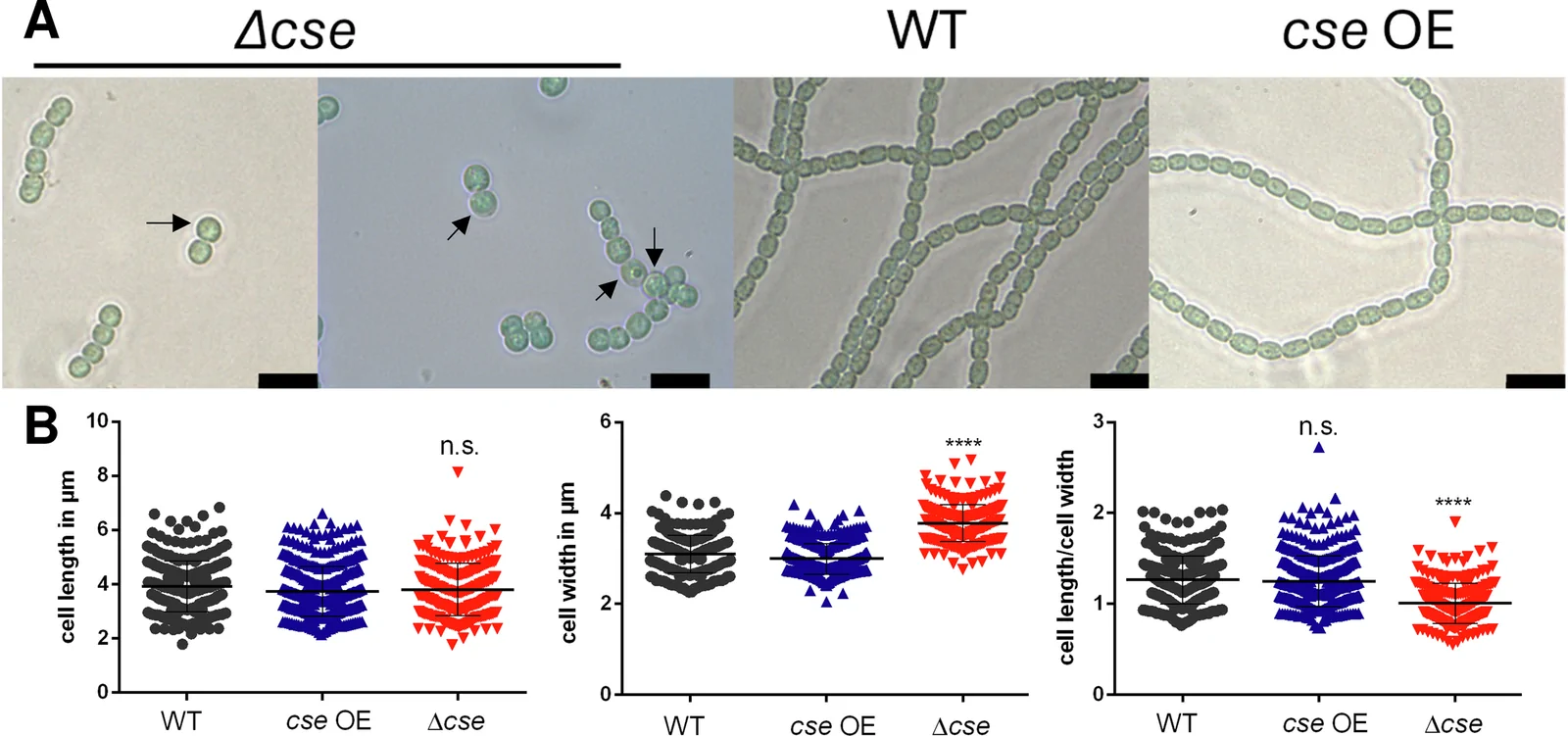
Ca^2+^-binding protein CSE controls cell shape and filament integrity. (**A**) Light microscopy of *Nostoc* wild-type (WT) strain and its derived *cse* knockout (Δ*cse*) and overexpression (*cse* OE) mutants, showing a strong fragmentation phenotype of the Δ*cse* mutant with irregular enlarged cells (indicated by black arrows). Bars, 10 µm. (**B**) Median cell length and cell width of the indicated strains in (**A**). Data are pooled from three independent biological replicates (exponentially growing) with standard deviation. Dimensions were measured from multiple filaments across 2–3 images per replicate (WT, *n* = 298 cells; Δ*cse*, *n* = 230 cells; *cse* OE 402 cells). Significance of Δ*cse* compared to WT was determined via Student’s *t* test: cell length, *P* = 0.17 (not significant); cell width, *P* < 0.0001 (****); *P* cell length/cell width ratio, *P* < 0.0001 (****). Whiskers represent standard deviation. n.s.: not significant. Source data are available online for this figure.

As the Δ*cse* mutant is suffering from heavy fragmentation, we decided to additionally check the cell–cell communication within the abnormal short filaments (down to only three cells; Fig. 4), which also feature unusually large cells (Fig. 5A). Here, almost 50% of the cells showed very slow cell–cell communication (slow increase) or even no cell–cell communication for 10–30% of the cells (Fig. 4), highlighting a central role for *cse* in controlling cell–cell communication.

**Figure EV5:**
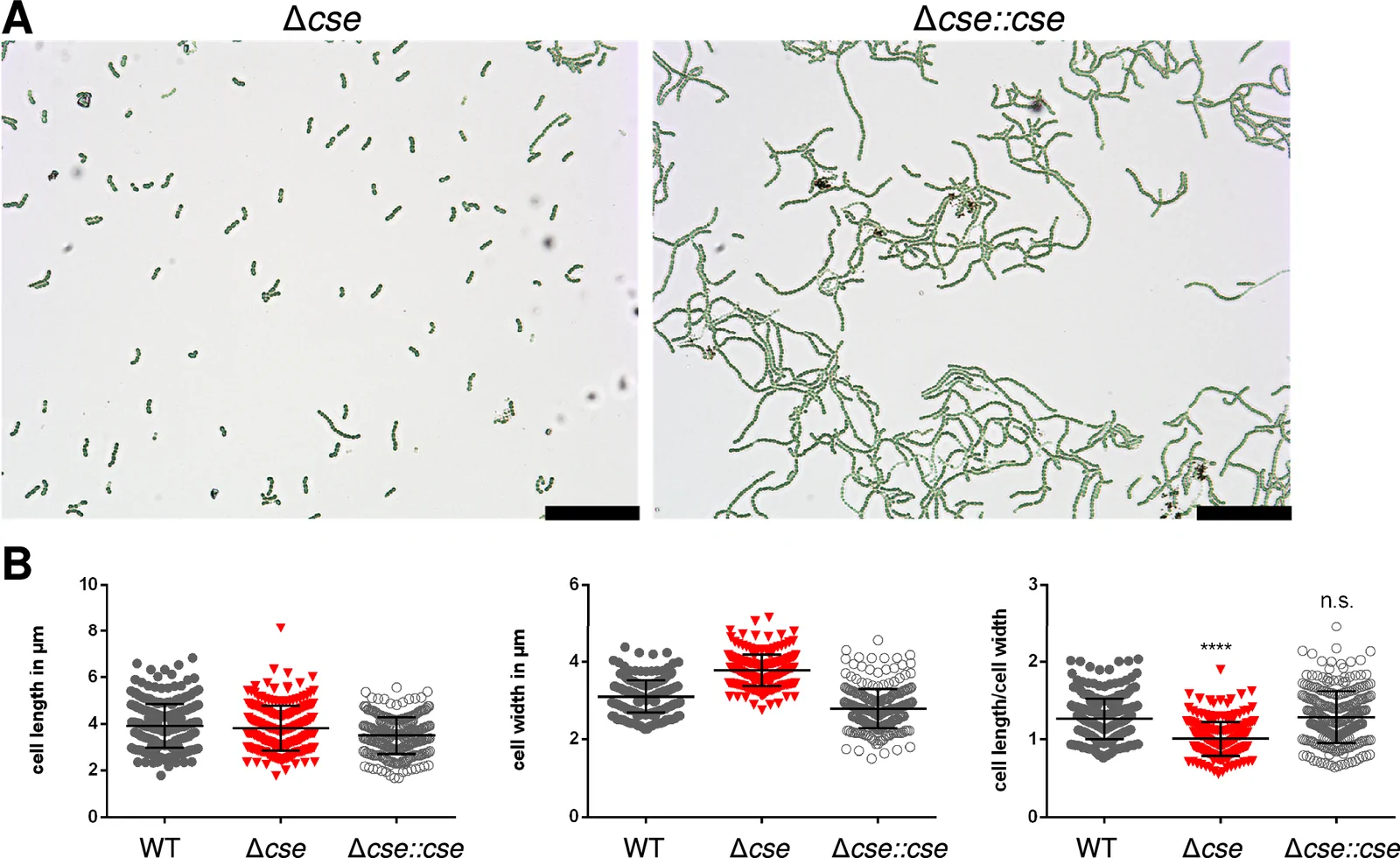
Ca^2+^-binding protein CSE controls cell shape and filament integrity. (**A**) Light microscopy of *cse* knockout (Δ*cse*) and the Δ*cse::cse* complementation strains, showing that the Δ*cse::cse* complementation strain exhibits wild-type (WT) like appearance with regular filaments (as in Fig. 5A) compared to the fragmentation phenotype of the Δ*cse* mutant. Bars, 100 µm. (**B**) Median cell length and cell width of WT and Δ*cse* strains (of Fig. 5B) compared to Δ*cse::cse* complementation strain. The Δ*cse::cse* complementation strain exhibits WT-like appearance compared to the Δ*cse* mutant. Data are pooled from three independent biological replicates (exponentially growing) with standard deviation. Dimensions were measured from multiple filaments across 2–3 images per replicate (WT, *n* = 298 cells; Δ*cse*, *n* = 230 cells; *cse* OE 402 cells). Significance of Δ*cse::cse* strain compared to WT was determined via Student’s *t* test: *P* cell length/cell width ratio, *P* < 0.0001 (****) for Δ*cse* strain and *P* = 0.47 (not significant) for Δ*cse::cse* complementation. Whiskers represent standard deviation. n.s. not significant. Source data are available online for this figure.

In multicellular cyanobacteria, cell size homeostasis is a fundamental physical trait, which impacts the overall cell fitness and cell–cell communication (Zeng et al, 2023). Our observation that the short filaments of Δ*cse* mutant encounters also irregular cell shape (Fig. 5A), motivated us to systematically check whether *cse* influences also the cell size. Interestingly, Δ*cse* cells exhibited significant cell size enlargement (particularly cell width), while *cse* OE mutant had comparable cell size parameters to the WT cells (Fig. 5B), further underscoring a vital role for CSE in multicellularity. In fact, the complemented Δ*cse*::*cse* strain also reversed the fragmentation (Fig. EV5A) and the cell size phenotypes to the wild-type appearance (Fig. EV5B).

### The abundance of SJs is drastically reduced in the Δ*cse* mutant

**Figure 6:**
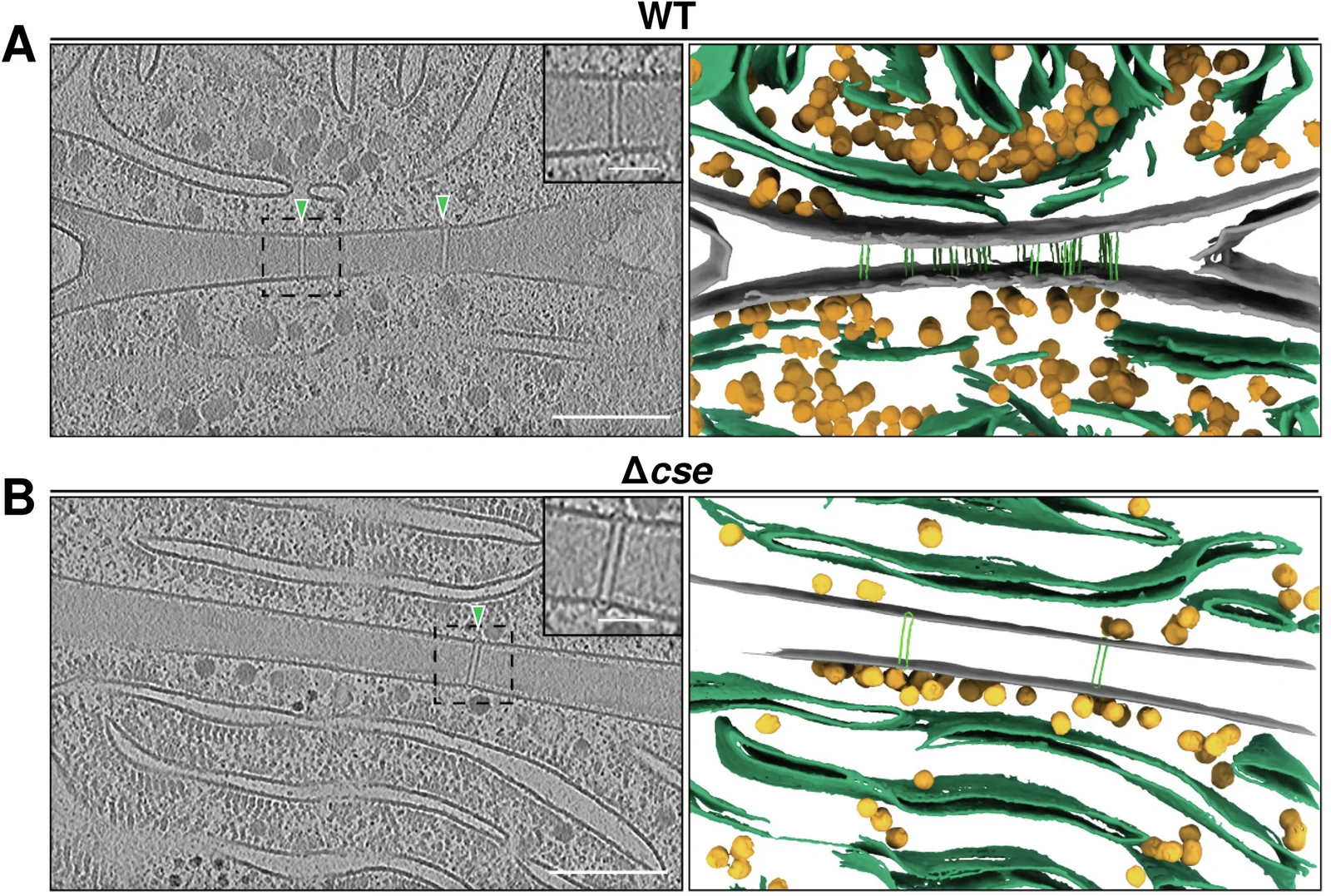
Cryo-ET reveals a reduced number of SJs in the Δ*cse* mutant. (**A**) Slice through a cryo-tomogram (shown is a 16-nm-thick slice) of a septum of a *Nostoc* WT filament (left), and the corresponding 3D segmentation (right) shows numerous SJs (green arrowheads) per septum. In this cryo-tomogram of a ~ 200-nm-thick lamella, 16 SJs were observed. A magnified view of one SJ is shown in the top right corner of the left panel. Scale bar cryo-tomogram, 200 nm. Scale bar magnified view, 50 nm. (**B**) Slice through a cryo-tomogram (shown is a 16-nm-thick slice) of a septum of a *Nostoc* Δ*cse* filament (left), and the corresponding 3D segmentation (right) shows a heavily reduced number of SJs (green arrowhead) per septum. In this cryo-tomogram of a 200-nm-thick lamella, only 2 SJs were observed. The overall architecture of SJs in the Δ*cse* mutant appears to be similar to WT (magnified view in the top right corner of the left panel). Another example of a cryo-tomogram of a septum of a *Nostoc* Δ*cse* filament can be seen in (Fig. EV6). Scale bars: 200 nm (cryo-tomogram slices) and 50 nm (magnified views).

**Figure EV6:**
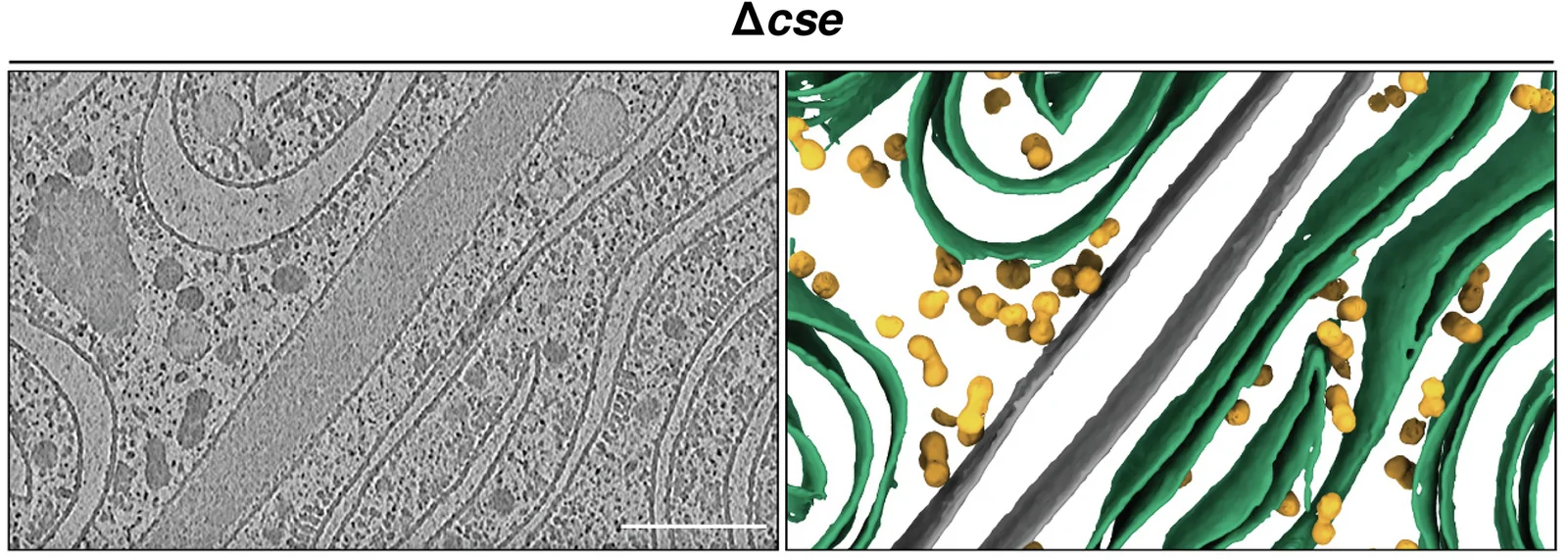
Cryo-ET reveals a highly reduced number of SJs in the Δ*cse* mutant. Slice through a cryo-tomogram (shown is a 16-nm-thick slice) of a septum of a *Nostoc* Δ*cse* filament (left), and the corresponding 3D segmentation (right) shows a heavily reduced number of SJs per septum. In this cryo-tomogram of a ~ 200-nm-thick lamella, no SJs were observed. In comparison, ~8 SJs/tomogram can be observed in WT filaments. Scale bar: 200 nm.

Next, we investigated the in situ architecture of the cell–cell communication machinery to understand how it is altered in the Δ*cse* strain. Cryo-ET of cryo-focused ion beam thinned *Nostoc* cells has proven to be a valuable tool to visualize and determine the in situ architecture of SJs (Weiss et al, 2019). For WT cells, we collected 22 tomograms of septa on 10 lamellae and observed SJs in 14 of them (64%) with an average of 8 SJs/tomogram (ranging from 1 to 18 SJs/tomogram; Fig. 6A; Movie EV1). This observation agrees well with previous calculations of ~10 SJs in a 200 nm lamella and with ~40–80 nanopores/septum (Weiss et al, 2019). With similar parameters, we collected 14 tomograms on 10 lamellae of Δ*cse* septa and surprisingly observed SJs in only 2 of them (14%) with only 2 or 4 SJs/tomogram (Figs. 6B and EV6; Movies EV2 and EV3). The overall architecture of SJs (cap, plug, and tube) in the Δ*cse* mutant appears similar to that of WT cells in the open state; however, their low abundance hindered subtomogram averaging to rule out more detailed structural changes. This striking reduction of SJs number in the Δ*cse* mutant explains the earlier results of slow cell–cell communication (Fig. 3B).

### Molecular basis for CSE-dependent control of cell–cell communication

Previous studies have suggested a correlation between cell–cell communication, filament integrity, and proper formation of nanopores in septal PG discs (Bornikoel et al, 2017; Nürnberg et al, 2015; Kieninger and Maldener, 2021; Merino-Puerto et al, 2011; Arévalo and Flores, 2021; Arévalo et al, 2021). In line with this, our cryo-ET data links the highly reduced number of SJs in the Δ*cse* mutant (Fig. 6) to the strong fragmentation phenotype (Fig. 5A) and the slow rate of cell–cell communication of the Δ*cse* strain (Fig. 3B). This may be simply due to a reduction in the number of nanopores. Another possibility is that even if a sufficient number of nanopores are built, they might not be fully mature yet, which might hinder the proper assembly of SJs and thereby negatively influence cell–cell communication.

**Figure 7:**
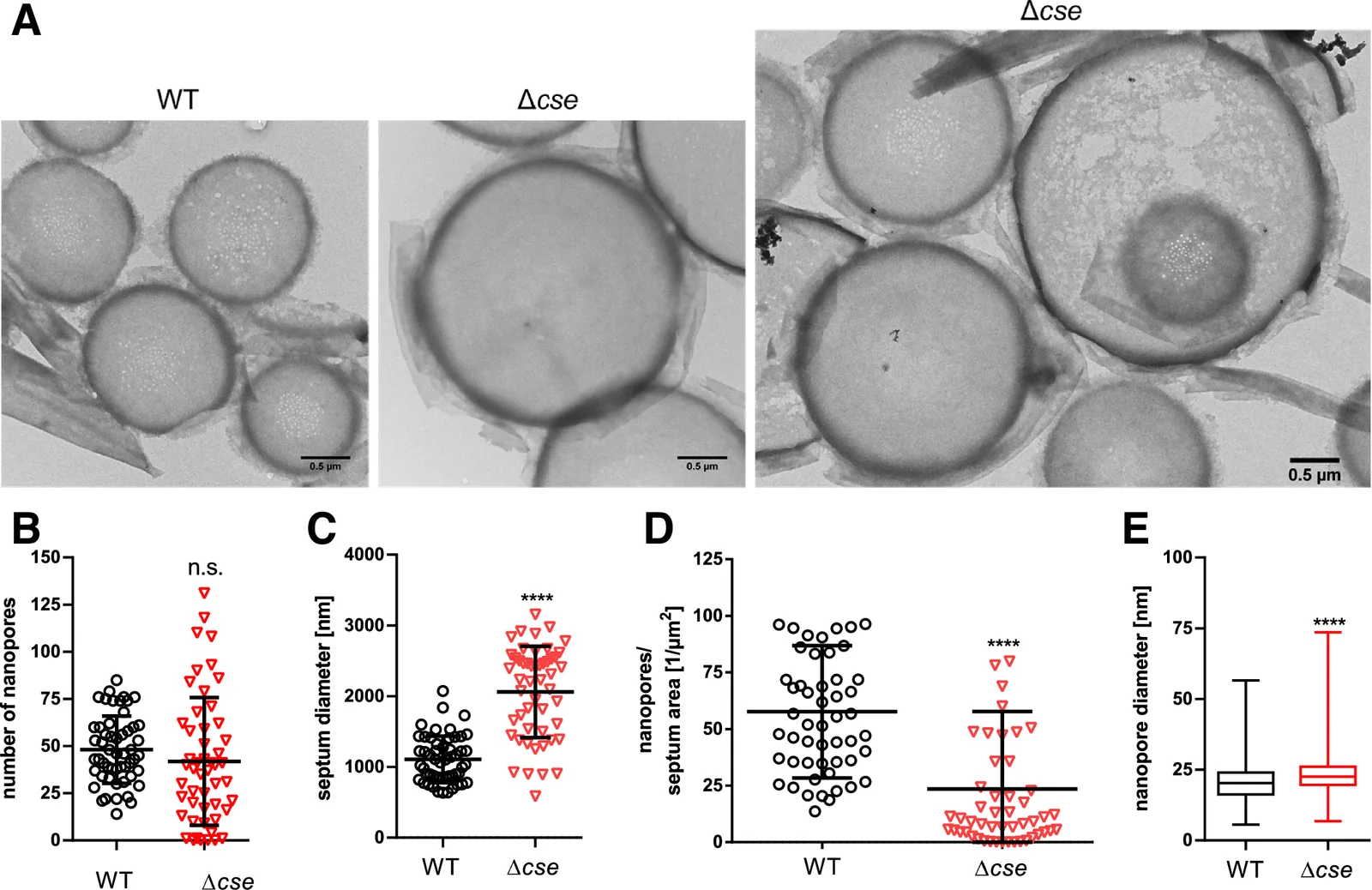
Isolated septal peptidoglycan discs of wild-type *Nostoc* (WT) and Δ*cse* strains. Data of the isolated septa analysis via electron microscopy. (**A**) Representative septal PG discs of the WT and Δ*cse* strains. (**B**) Number of nanopores with overall mean and standard deviation (SD) of WT and Δ*cse* strains. (**C**) Septum diameter in nm with mean and SD of WT and Δ*cse* strains. (**D**) Ratio of the nanopore number per septum area of each data point with the overall mean and SD. (**E**) Box plot of nanopore diameter in nm for WT and Δ*cse* strains. The box center indicates the median, while the lower and upper bounds of the box represent the 25th and 75th percentiles, respectively. Whiskers indicate the minimum and maximum values. WT: Median = 20.3 nm (IQR: 16.3–24.0 nm), Range = 5.6–56.6 nm. Δ*cse*: Median = 22.5 nm (IQR: 19.6–26.1 nm), Range =  6.8–73.6 nm. The graphs (**B**–**E**) represent the pooled data of biological triplicates with standard deviation. Significance: ****P value < 0.0001 in Student’s *t* test, in comparison to WT. Whiskers: standard deviation. n.s. not significant. Source data are available online for this figure.

To explain the Δ*cse* phenotype (Fig. 3B; Fig. 6) mechanistically and to obtain further evidence of a functional link between CSE and cell–cell communication, we determined the efficiency of nanopore array formation in the Δ*cse* mutant. For this purpose, we isolated the septal discs of the WT and Δ*cse* strains and quantified the nanopore formation using negative staining EM (Fig. 7). It became evident that the Δ*cse* mutant possesses a huge variation in the number of nanopores compared to WT cells (Δ*cse*: 41.9 ± 34.9; WT: 48.1 ± 17.8), with some septa almost free from any nanopores (Fig. 7A,B), explaining our cryo-ET results (Figs. 6 and EV6). Interestingly, we additionally observed an altered septum diameter (Fig. 7C). The diameter of septal discs in WT cells agreed with the previously published data (Arévalo and Flores, 2021), while the septa of Δ*cse* cells appeared to be greatly enlarged with sizes almost double those of the WT cells (Δ*cse*: 2062 ± 645 nm; WT: 1107 ± 329 nm; Fig. 7C), implying an immaturation of Δ*cse* septa. When we compared the number of nanopores to the increased septum diameter it became apparent that the Δ*cse* septal discs harbored fewer nanopores per septum area of 1/µm^2^ (Fig. 7D). In this case, the increased septal area (Fig. 7C) does not lead to an increased number of nanopores, further supporting the immaturation of Δ*cse* septa and explaining the slow rate of cell–cell communication (Fig. 3B). The WT exhibited a ratio of 55 nanopores per septum area of 1/µm^2^, whereas Δ*cse* possessed only 26 nanopores per septum area of 1/µM^2^, which is approx. 47% of the WT cells. Also, it became more obvious that the Δ*cse* mutant possesses a massive discrepancy with some septa without any nanopores or just very few (Fig. 7D). The nanopore diameter was rather similar for both strains (Δ*cse*: 22.5 nm; WT: 20.3 nm; Fig. 7E). Overall, the Δ*cse* mutant exhibited a clear defect in nanopore formation, supporting a role for CSE in regulating cell–cell communication. Remarkably, several mutants, which are involved in SJs (e.g., FraD) or nanopore arrays formation (e.g., AmiC), showed a fragmentation phenotype with enlargement in the septal discs and/or a reduction in the number of nanopores (Bornikoel et al, 2017; Kieninger et al, 2022; Arévalo and Flores, 2021), similar to that observed for Δ*cse*, further boosting an essential role for Ca^2+^-binding protein CSE in regulating cell–cell communication.

As the EF-hand protein family (e.g., calmodulin), to which CSE belongs, is known also to exert their regulatory functions via protein-protein interactions (Walter et al, 2019; Heizmann, 2019), we wondered whether CSE could bind to yet unknown targets in a Ca^2+^-dependent manner, thereby affecting septal discs and nanopore arrays. For this purpose, we performed several pulldown experiments (in the presence or absence of 2 mM Ca^2+^) using immobilized Strep-tagged CSE protein on streptavidin magnetic beads to identify potential targets of CSE. As negative controls, empty beads were used to exclude the proteins, which could bind unspecifically to the beads. Unrelated Strep-tagged PII protein (Forchhammer and Selim, 2020) was also used as an additional control to exclude the unspecific proteins. The pulldown experiments identified several potential targets of CSE (summarized in Appendix Table S3). Few targets were identified in the presence of 2 mM Ca^2+^ compared to the absence of Ca^2+^ (Appendix Fig. S2 and Appendix Fig. S3). Next, we applied a strict cut-off to sort for promising CSE-targets, requiring a minimum score of 20 and enrichment in CSE-based pulldowns compared to at least two controls. This analysis identified three potential CSE-targets, two of which were identified in the absence of Ca^2+^ (phosphate binding protein All2358 and amidase AmiC3 All1140), suggesting that Ca^2+^-binding to CSE disrupts the complex formation, while the third target (Ca^2+^-dependent Serralysin-like metalloprotease Alr3659) was enriched regardless of Ca^2+^ presence. Of these targets, AmiC3 appeared as a promising target due to its involvement in nanopore formation and controlling the diameter of nanopores (Zheng et al, 2017). To validate and test the influence of Ca^2+^ on the CSE-AmiC3 interaction by an independent method, we carried out physical interaction assays using purified proteins. When CSE was immobilized on streptavidin beads, coelution of His-tagged AmiC3 could be detected, while the presence of 2 mM Ca^2+^ in the buffer weakened the CSE-AmiC3 interaction, further validating the pulldown experiments (Appendix Fig. S2B). To gain further insight into CSE-AmiC3 complex formation, we studied the physical CSE-AmiC3 interaction using mass photometry (Appendix Fig. S2C). Compared to AmiC3 alone, the CSE-AmiC3 interaction showed a mass increase ( ≈ 10 kDa of CSE) corresponding to complex formation (Appendix Fig. S2C). Again, the presence of Ca^2+^ weakened the complex formation as the mass count of CSE-AmiC3 interaction is reduced by almost 50% in the presence of 2 m M Ca^2+^ compared to the Ca^2+^-free complex.

## Discussion

Filamentous cyanobacteria are among the oldest multicellular organisms on Earth, rendering them crucial for understanding the evolutionary aspects of cell–cell communication. Cyanobacterial multicellularity includes cell differentiation and cell–cell communication for exchanging nutrients and signaling molecules between neighboring cells. We previously showed that the cyanobacterial cell–cell communication machinery is composed of nanopore arrays drilled by amidases and SJs tubes passing between the PG layers of two adjacent cells (Weiss et al, 2019). A strong fragmentation phenotype of cyanobacterial filaments is often associated with disrupting genes involved in controlling cell–cell communication machinery (Kieninger et al, 2022; Kieninger and Maldener, 2021). As the Δ*cse* mutant is suffering from a severe fragmentation phenotype (Walter et al, 2020) (Fig. 5A), we conjectured that the Ca^2+^ via the CSE protein may be the missing link controlling the cell–cell communication machinery in multicellular cyanobacteria. Here, we revealed that the Ca^2+^ regulates the cell–cell communication machinery, as indicated by a slow communication rate for the Δ*cse* mutant (Fig. 3B; Fig. 4). This is driven by a decrease in nanopore numbers (Fig. 7), which subsequently reduces the active SJs, rather than influencing the SJ’s architecture. Another plausible explanation is the influence of CSE on cell volume relative to the number of SJs and nanopores, as the Δ*cse* mutant possesses an increase in both cell width (Fig. 5B) and septum diameter (Fig. 7C), which might affect the diffusion rate of the FRAP fluorophore, calcein. However, both possibilities do not exclude each other, but rather support a vibrant role for Ca^2+^ via its receptor CSE.

The signals, controlling cyanobacterial cell–cell communication machinery, were largely unknown. To our knowledge, CSE is the first Ca^2+^-buffer protein known to regulate cell–cell communication. This highlights Ca^2+^-signaling via CSE as a key point in cyanobacterial multicellularity, influencing not only filament integrity (Fig. 5), cell dimensions, heterocyst differentiation and photosynthesis (Walter et al, 2019, 2020), but also the cell–cell communication (Fig. 3; Fig. 4). The diverse phenotypic effects associated with *cse* mutation appear to stem from a perturbation of Ca^2+^-homeostasis, a scenario that aligns with its function as a Ca^2+^-buffer protein, but does not exclude the possibility that CSE is also directly involved in multiple signaling pathways. The very recent discoveries of cellular junctions regulated by long-range Ca^2+^-signaling or directly by Ca^2+^ ions (Benedetti et al, 2025; Flores et al, 2025), further support our findings and suggest that the regulation of cellular junctions through Ca^2+^-signaling might be an evolutionarily conserved principle between organisms that diverged at least a billion years ago. This also suggests that this regulatory principle arose earlier in multicellular prokaryotes before it appeared in eukaryotes.

Our results revealed an enlargement in the Δ*cse* septa and an alteration in the nanopore numbers (Fig. 7), similar to that observed previously for several mutants encoding for key components of the cell–cell communication machinery (e.g., Δ*fraD* and Δ*amiC*) (Bornikoel et al, 2017; Kieninger et al, 2022; Weiss et al, 2019; Arévalo and Flores, 2021). The Δ*cse* mutant showed a remarkable variation in the number of nanopores, with some septa completely free of nanopores or with just very few (Fig. 7). In most mutants where the number of nanopores is drastically reduced, the fragmentation of the filament frequently occurs. This, together with the abnormally large septa and the great influence on cell volume (Fig. 5), hints that the Ca^2+^-binding protein CSE is required for the correct formation of the septal discs and nanopore arrays.

Large septal discs are usually observed in young, immature, and divided cells, explaining the few nanopores observed. Given the strong fragmentation phenotype of the Δ*cse* mutant and its negative influence on the nanopore arrays and septa, it’s possible that CSE additionally regulates cell division, septal plane, or cell wall maturation via the cellular amidases (AmiCs), which are involved in drilling and maturation of nanopores. *Nostoc* possesses three amidases, which are all required for proper intercellular communication. While AmiC1 and AmiC2 are involved in nanopores formation, AmiC3 regulates the nanopore size (Bornikoel et al, 2017; Lehner et al, 2013; Zheng et al, 2017). Nevertheless, it’s largely unknown how the cell wall amidases are regulated, therefore it’s possible that CSE interacts directly with one or more of AmiCs, similar to LytM, which interact with AmiC1 and enhances its activity (Bornikoel et al, 2018). Indeed, our results showed AmiC3 (among other targets) as an interacting partner of CSE (Appendix Fig. S2), however, the relevance of CSE-AmiC3 interaction will need further investigations to understand the influence of CSE on AmiC3. CSE interaction might regulate the amidase activity or its cellular localization, thereby influencing on the septal discs, the nanopore arrays, and cell–cell communication. Other open questions are: whether the Ca^2+^-dependent metalloprotease (encoded by *alr3659*) is a true interacting partner of CSE, and what are the cellular functions of this Serralysin-like metalloprotease, particularly regarding the Ca^2+^-signaling?

As CSE acts as Ca^2+^-buffer protein and Ca^2+^ is known to operate more broadly in metabolic signaling, it is also possible that free Ca^2+^ ions directly control the amidase activity, induce conformational changes on the SJs components, or influence the levels of protein transcriptionally or translationally. Notably, external Ca^2+^ supplementation had no influence on the communication rate, consistent with the tight control of free cytoplasmic Ca^2+^ ions (at nM levels) through Ca^2+^ pumps and Ca^2+^-buffer proteins (Mantovani et al, 2023).

Our structural analysis supports the notion that CSE is a Ca^2+^-buffer protein. In the absence of divalent ions, CSE is substantially unfolded, both Ca^2+^ (and to some extent Mg^2+^) ions can induce folding, supporting the high specificity of CSE towards Ca^2+^-binding. While Ca^2+^ concentrations are strictly controlled at nM levels, Mg^2+^ is one of the most abundant metal ions in the cytoplasm; therefore, a true apo-form of CSE may not exist in the cell. Thus, CSE likely exists in a partially folded ‘waiting’ state at low Ca^2+^ concentrations, and the buffering capacity of CSE is likely controlled by the relatively high affinity for Ca^2+^ compared to Mg^2+^ ions (Walter et al, 2019). CSE possesses two EF-hand Ca^2+^-binding motifs responsible for sequestering free cytoplasmic Ca^2+^ ions. Similarly, several EF-hand Ca^2+^-binding proteins were shown to act as Ca^2+^-buffer proteins, further supporting our finding. For example, *Pseudomonas aeruginosa* encodes an EF-hand Ca^2+^-buffer protein, EfhP (Sarkisova et al, 2014), while the filamentous actinobacterium *Streptomyces coelicolor* harbors four EF-hand Ca^2+^-binding proteins (CabA, CabB, CabC, and CabD), suggesting a role for Ca^2+^ EF-hand proteins in multicellularity (Domínguez et al, 2015; Wang et al, 2008; Yonekawa et al, 2005; Zhao et al, 2010). Filamentous cyanobacteria possess another conserved Ca^2+^-binding protein, known as CcbP (Zhao et al, 2005; Hu et al, 2011). In contrast to CSE, CcbP seems to be implicated mainly in heterocyst differentiation, and Ca^2+^-binding does not induce strong conformational changes in its structure. CcbP captures free Ca^2+^ ions via a noncanonical high-affinity binding site with an α-turn-β region and a weak site with an EF-hand-like motif.

Though only two Ca^2+^-binding proteins are known and analyzed in *Nostoc*, there are vast majority of putative EF-hand proteins in cyanobacteria. The CSE protein appears to be very important for multicellularity, as—with a few exceptions—it is only found in filamentous cyanobacteria and plays important roles in regulating filament integrity, heterocyst differentiation, and cell–cell communication machinery (Walter et al, 2019).

## Methods

**Table:** Reagents and tools table

Reagent/resource	Reference or source	Identifier or catalog number
**Experimental models**		
*Nostoc* sp. PCC 7120	Forchhammer Lab. (University of Tübingen, Germany)	N/A
Δ*cse*	Walter et al, 2019	N/A
*cse* oe	This study	N/A
Δ*cse::cse*	This study	N/A
pMBA224 (*cse-sfgfp*)	Gifted by Manuel Brenez-Alvarez (University of Freiburg, Germany)	N/A
*E. coli* BL21 (DE3)	Sigma-Aldrich	N/A
**PCR primers**		
See Appendix Table S5		
**Recombinant DNA**		
*pBG2089*	Walter et al, 2019; Gifted by Peter Gollan (University of Turku, Finland)	N/A
*pASK-IBA5*	IBA Lifesciences GmbH	N/A
*pET15b*	Addgene	N/A
**Chemicals, enzymes, and other reagents**		
^13^C-glucose		
^15^N-ammonium chloride		
IPTG	Sigma-Aldrich	CAS No.. 367-93-1
Trizma^®^ base	Sigma-Aldrich	CAS No.. 77-86-1; T1503
NaCl	Fisher Scientific	CAS No.. 7647-14-5, Art.Nr.. 10123443
Bg11	See Appendix Table S4	
BD™ Difco™ Agar	Becton Dickinson	REF 214010
Q5 polymerase	NEB	M0491
Calcein-AM	Thermo fisher	C1430
DMSO	Fluka Chemika	41648
CaCl_2_	Fisher Scientific	CAS No. 10043-52-4,
SDS	Sigma-Aldrich	75746
Na_3_PO_4_	Bernd Kraft	15312.3600
α-Chymotrypsin	Sigma-Aldrich	CAS No. 9004-07-3; C4129
Vancomycin, BODIPY FL conjugate	Invitrogen	V34850
UV-irradiated formvar/carbon film-coated copper grids	Electron Microscopy Sciences	FCF200-CU-SB
Uranyl acetate	N.A.	N.A.
NEB Q5 site-directed mutagenesis kit	NEB	E0554S
Anhydrotetracycline	Thermo Scientific	Cat. 233131000
Strep-Tactin®	IBA Lifesciences GmbH	N/A
Chelex 100	Sigma (200–400 mesh)	95621
MagStrep® “typ3” XT beads	IBA Lifesciences GmbH	17302981
NaCl	Fisher Scientific	CAS No. 7647-14-5
Strep-BXT Biotin Elution Buffer	IBA	N/A
4–15% SDS Q-PAGE™ TGN Precast Gel	SMOBIO Technology, Inc	N/A
Blauer Jonas	German Research Products Ltd	GRP1
Silicon gaskets	3 mm diameter × 1 mm depth, Grace Bio-Labs, Bend, OR, USA	
Coverslips	Marienfeld, Germany No. 1.5H 24 × 5	
Glow-discharged copper EM grids	R2/2, Quantifoil	
Teflon sheet		
FIB milling autoloader-grids	Thermo Fisher Scientific, Waltham, MA	
8-nm-thick layer of tungsten		
BioContinuum imaging filter slit width 20 eV		
**Software**		
AlphaFold2.0		
GraphPad Prism		
ImageJ		
Excel		
Zeiss Zen Blue software		
MicroCal PEAG-ITControl Software (Malvern, UK)		
AcquireMP software		
DiscoverMP software		
SerialFIB software package		
SerialEM and PACEtomo (Eisenstein et al, 2023) software packages.	Mastronarde, 2005, 2003 Eisenstein et al, 2023	
IMOD package	Kremer et al, 1996; Mastronarde, 2008	
IsoNet	Liu et al, 2022	
Dragonfly	Heebner et al, 2022	
ChimeraX		
**Other**		
Ni-NTA columns		
Äkta-system		
Bruker Avance-III spectrometers		
Zeiss LSM 800 confocal microscope		
Zeiss LSM 880 confocal microscope		
Sonifier	Branson Sonifier 250	
Sonfier water bath	RK100, Bandelin electronic, Berlin	
Philips Tecnai 10 electron microscope		
Rio Camera	Gatan	
MICROCal PEAQ-ITC SYSTEM microcalorimeter	Malvern, UK	
TwoMP mass photometer	Refeyn, Oxford, UK	
Vitrobot plunge-freezing robot	Thermo Fisher	
Zeiss Crossbeam 550 FIB-SEM dual-beam instrument	Carl Zeiss Microscopy, Oberkochen	
40° pre-tilted grid holder	Leica Microsystems GmbH, Vienna, Austria	
VCM loading station	Leica Microsystems GmbH, Vienna, Austria	
VCT500 cryo-transfer system	Leica Microsystems GmbH, Vienna, Austria	
ACE600 cryo-sputter coater	Leica Microsystems GmbH, Vienna, Austria	
copper-band cooled mechanical cryo-stage	Leica Microsystems GmbH, Vienna, Austria	
Titan Krios 300 kV FEG transmission electron microscope	Thermo Fisher	
K3 direct electron detector	Gatan	

### NMR spectroscopy

For NMR structure determination, N-terminally His_6_-tagged CSE protein was expressed in minimal medium containing ^13^C-glucose and ^15^N-ammonium chloride at 37 °C by induction with IPTG. The purification process involved Ni-NTA chromatography followed by size-exclusion chromatography using an Äkta system as described previously (Walter et al, 2019). The protein was eluted in a 20 mM Tris-HCl buffer (pH 7.9) containing 100 mM NaCl.

NMR experiments were conducted at 293 K on 600 MHz Bruker Avance-III and 800 MHz Bruker Avance-III spectrometers. Backbone sequential assignments were made using standard triple-resonance experiments. Note that these were only possible for a freshly prepared ion-free sample that was subsequently saturated with 2 mM CaCl_2_. As the CoMAND structure determination protocol employs a ^13^C-edited 3D-CNH-NOESY, the aim of the side chain assignment process was to achieve the most complete ^13^C assignment possible. To this end, we used dedicated TOCSY-based experiments for both aliphatic and aromatic side chain assignments.

Structure determination of Ca^2+^-saturated CSE was performed using the CoMAND protocol (ElGamacy et al, 2019). The input data for this protocol were derived from a 3D-CNH-NOESY spectrum (Diercks et al, 1999) obtained at 800 MHz (mixing time 120 μs). The processed 3D matrix was first broken into a set of 1D vectors perpendicular to assigned ^15^N-HSQC positions, such that each contains peaks to one ^15^N-bound proton. The CoMAND method is based on selecting conformers from a pool in order to best explain these experimental vectors (strips). This is judged by a quantitative R-factor reflecting the match between experimental and back-calculated data. As each strip is associated with a single ^15^N-bound proton, the CoMAND R-factor is calculated on a per-residue basis.

The starting point for the CoMAND protocol is a model of the folded protein. For this purpose, we chose the AlphaFold2.0 model, which has very high confidence scores on the level of the global fold. In order to sample conformational diversity, we used standard simulated annealing protocols in XPLOR-NIH (Schwieters et al, 2006, 2003), using generic distance and dihedral angle restraints generated from the starting model. These runs have the advantage of efficient sampling of conformational space for loops and unstructured regions of the protein. These simulations were run with Ca^2+^-bound via geometric restraints based on the canonical EF-hand binding mode and pentagonal bipyramidal Ca^2+^ geometry. A total of 50 structures were calculated, from which we selected the best 8 models, based on their overall R-factor. The selected structures were then used as individual starting points for unrestrained molecular dynamics simulation runs in explicit solvent using NAMD 2.12 (Phillips et al, 2020) with an underlying CHARMM36 forcefield.

For the first unrestrained MD runs, we sampled a total of 4000 frames, from which we again selected based on R-factor. In this case, we selected at the per-residue level; i.e., a set of MD frames was selected to optimize the R-factors for each residue for which strips were available. This optimization provides detailed information on the conformational distributions for each residue. In order to investigate the conformational diversity of residues expected to contact the calcium ion, we carried out these simulations without bound calcium. It should be noted that conformations consistent with calcium binding are under-sampled in such simulations, due to unfavorable charge distributions. Nevertheless, sufficient information on the distributions was obtained for each residue to confirm the canonical calcium binding mode. The sets of frames collated for each residue were used to compile distance and dihedral restraints that effectively replace those based on the original model. These were used in a second XPLOR-NIH run to generate a new set of 400 structures.

At this stage, it is expected that optimization based on single residues should explain the experimental data very well, in most cases approaching the boundary represented by experimental noise and the accuracy of back-calculation. For some residues, this was not the case, suggesting that the conformations present in the original AlphaFold2.0 model were not sufficient to explain the data. These included two backbone polymorphisms that are typically not well predicted by static AlphaFold2.0 models. In this case, we employed exhaustive grid searches to identify any upsampled conformers and built models containing the alternate conformation in XPLOR-NIH using appropriate distance and dihedral restraints. A set of 12 structures respecting these polymorphisms was compiled as the starting point for a final round of MD sampling, with each used to seed a short trajectory at 310 K in explicit solvent, in this case conducted with calcium modeled into the binding sites. We selected the final ensemble from this pool of 2460 frames.

### Cyanobacterial strains and verification of segregation of Δ*cse*

*Nostoc* sp. PCC 7120 was used as the wild-type (WT) strain. The Δ*cse* strain was gifted from Peter Gollan's laboratory (University of Turku, Finland). In this strain, the gene coding for CSE, *asr1131*, was replaced by a neomycin resistance cassette (Walter et al, 2020). The overexpression and complementation strains were generated as described previously (Walter et al, 2019, 2020) using *cse* coding sequence under its native promoter within the low-copy number plasmid *pRL1342*. The strain expressing CSE-sfGFP fusion was gifted from Manuel Brenez-Alvarez (University of Freiburg, Germany). *Nostoc* sp. PCC 7120 and the *cse* mutants were cultivated in blue-green media with sodium nitrate (BG11; shown in Appendix Table S4) (Rippka et al, 1979). Liquid cultures were grown under agitation at 125 rpm and plate cultures were grown on solidified BG11 with 1.5% (w/v) Difco agar (Becton Dickinson) at 28 °C and constant illumination at 30–40 µE m^−2^ s^−1^. The full segregation of the Δ*cse* strain was tested by colony PCR (Appendix Fig. S4) using high-fidelity polymerase Q5 (NEB) and different primer sets (Appendix Table S5).

### Fluorescence recovery after photobleaching (FRAP) analysis for measuring cell–cell communication

The fluorescent dye calcein was used to assess cell–cell communication as stated earlier (Merino-Puerto et al, 2011; Mullineaux et al, 2008). The method was further modified according to (Weiss et al, 2019) to test the cell–cell communication under different calcium chloride concentrations (0 mM, 0.25 mM, 1.25 mM CaCl_2_). The day before FRAP measurements, cells from liquid preculture (OD_750_ 0.6–1, BG11) were washed three times in 1 ml BG11 medium with respective calcium chloride concentration and spread on 1.5% (w/v) agar plates with the respective calcium chloride concentration. On the day of measurement, the cells were washed three times in medium and resuspended to an OD_750_ = 2.4 in 250 µl. The fluorescent dye calcein-AM (stock 1 mg∙ml^−1^ in DMSO, Thermo Fisher) (4–6 µl) was added. The samples were incubated for 90 min at 28 °C in the dark under constant gentle agitation and washed three times at 1700×*g* for 2 min with medium afterward. The cells were resuspended in 500 µl BG11 medium and further incubated for 90 min. For FRAP, 10 µl of each sample was spotted onto a BG11 agar plate with the respective calcium chloride concentration. The dried samples were studied with a Zeiss LSM 800 confocal microscope. For normal cells, the middle cell of a filament consisting of at least five equally dyed cells was bleached. For the Δ*cse* strain, shorter filaments (3 cells) with deviating cell size were assessed as well. Before bleaching, five pictures were taken. Pictures at 1.1 s intervals were taken for 60-100 s to record the fluorescence recovery in the bleached cells. Next, we used the GraphPad Prism and ImageJ programs to analyze the relative fluorescence in the bleached cells over time, as described previously (Weiss et al, 2019; Kieninger, 2021). For cells showing “full recovery” the recovery rates were calculated using a non-linear regression. Example curves for “full recovery” and “sigmoidal increase” are visualized in (Fig. EV4A).

To make sure that there is no overlap between calcein-AM emission and autofluorescence of bleached cells, a lambda scan was recorded using a Zeiss LSM 880 confocal microscope. *Nostoc* cells (unstained or stained with calcein-AM) were excited with a wavelength of 405 nm, and emission was captured in 32 pictures from 415 nm to 691 nm (step size of 8.625 nm), which were assembled using the Zeiss Zen Blue software. Lambda scan analysis revealed no overlap between calcein-AM emission and cyanobacterial autofluorescence (Fig. EV4C,D), supporting that the fluorescence recovery in FRAP experiments arises specifically from calcein-AM recovery.

### Septa isolation and transmission electron microscopy

To assess the properties of the septal disc, the septa were isolated according to (Kieninger, 2021; Kühner et al, 2014). Only filtered (0.22 µm) solutions were used. Cells from exponentially growing liquid cultures of *Nostoc* strains were spread on BG11 plates and incubated for 3 days under standard growth conditions. One third to one half of the cell material was scratched and resuspended in 1 ml ultrapure water and centrifuged at 10,000×*g* for 5 min. For fragmentation and mechanical disruption of the membranes, the cell pellets were resuspended in 700 μl 0.1 M Tris buffer (pH 6.8) and sonicated (duty care = 50, output control=1) for 2 min. Subsequently 300 µl of 10% SDS was added for denaturation. To melt the membranes, the samples were incubated at 99 °C and 300 rpm for 25 min. After centrifugation at 10,000×*g* for 5 min the pellet was washed twice with 1 ml ultrapure water. The peptidoglycan sacculi were mechanically degraded by incubation in a sonification water bath for 30 min. The samples were again centrifuged at 10,000×*g* for 5 min. The pellet was resuspended in 1.5 ml 50 mM Na_3_PO_4_ solution (pH 6.8) and incubated for 3 h with 15 μl α-Chymotrypsin (300 µg) added for enzymatic proteolysis. Further 15 μl α-Chymotrypsin (300 µg) were added, and the reaction mix was incubated overnight. Via incubation at 99 °C for 3 min the enzyme was inactivated, and the mix was centrifuged. The quality of the sample was assessed in the fluorescence microscope by the addition of 1% (v/v) fluorescent vancomycin (Vancomycin, BODIPY FL conjugate, 100 μl/ml in DMSO, Invitrogen). If required, additional sonication steps for 30–40 s followed. After centrifugation, the pellet was resuspended to a final volume of 300–500 µl from which 10 µl were applied to UV-irradiated formvar/carbon film-coated copper grids. After 30–60 min incubation, the grids were washed with ultrapure water and dried with filter paper before incubation with uranyl acetate (1% in ultrapure water) for 1 min. Dried grids were assessed with transmission electron microscopy using a Philips Tecnai 10 electron microscope at 80 kV connected to a Rio Camera (Gatan) for imaging. Transmission electron micrographs were analyzed as described in (Kieninger, 2021).

### Protein overexpression and purification

The plasmid overexpression of N-terminally Strep-tagged wild-type CSE protein was constructed using PCR with the oligonucleotides (#115 and #116). The *cse* PCR product, together with a short linker sequence, was fused into a linearized ***pASK-IBA5*** (IBA Lifesciences GmbH) plasmid using Gibson assembly (Selim et al, 2021b, 2018). The overexpression plasmid for the CSE triple variant (D54/56/58 A) was created based on the wild-type plasmid using the NEB Q5 site-directed mutagenesis kit and the oligonucleotides (#148 and #149), allowing substitution of aspartate with alanine at the positions 45, 56, and 58 of the CSE sequence. The primers used in this study are listed in (Appendix Table S5).

*E. coli* BL21 cells were used for the overexpression of CSE strep-tagged proteins. The overexpression was induced by adding 200 µg/l anhydrotetracycline for overnight at 20 °C. The protein was purified from the cell lysate using a peristaltic pump and affinity chromatography based on Strep-Tactin® (IBA Lifesciences GmbH).

### Isothermal titration calorimetry (ITC)

MICROCal PEAQ-ITC SYSTEM (Malvern, UK) microcalorimeter was used as described previously (Walter et al, 2019; Lapina et al, 2018; Mantovani et al, 2024) to calculate the binding affinity of Ca^2+^-binding to wild-type CSE or its triple mutant variant (D54/56/58A) proteins. Purified proteins were extensively dialyzed in 20 mM Tris-HCl buffer (pH 7.9) with Chelex 100 (Sigma) to remove any metal ion contamination (Walter et al, 2019). For determining ligand binding isotherms, 66 or 132 μM protein solutions (monomeric concentration) were titrated against 1 mM of Ca^2+^. The binding isotherms were calculated from the received data and fitted using one set of binding sites model with the MicroCal PEAG-ITControl Software (Malvern, UK) to calculate the dissociation constant K_D_ (Selim et al, 2018). All titrations were performed in duplicate. The K_D_ value for the strep-tagged wild-type CSE protein (1.8 µM) was comparable to the previously measured His-tagged CSE protein with 1.4 µM (Walter et al, 2019, 2020).

### Pull-down with crude cell extracts from *Nostoc* sp. PCC 7120 cells

To find possible interaction partners of CSE, a mass spectrometry-based pull-down approach was used as described previously (Selim et al, 2021a) using Strep-tagged CSE protein in the presence or absence of 2 mM CaCl_2_. 15 µM purified CSE (used as a bait) was incubated for 20 min at 28 °C to 150 µl magnetic MagStrep® “typ3” XT beads (IBA Lifesciences GmbH). Then the crude cell extract from the Δ*cse* mutant was added to the CSE-bound to magnetic MagStrep beads and further incubated for 1 h. Unrelated Strep-tagged PII protein was used as a negative control to exclude the unspecific proteins. Also, empty MagStrep beads were used as an additional control to exclude the proteins, which could unspecifically bind to the beads. The empty MagStrep beads were challenged with *Nostoc* cell extract in the presence or absence of 2 mM CaCl_2_. After extensive washing, the bound proteins were eluted and sent to mass spectrometry-based proteomics analysis. Label-free quantification was used to calculate intensities and iBAQ values that give semiquantitative quantifications of protein enrichment. The number of uniquely identified peptides/proteins, sequence coverage, and score were considered to select proteins of interest.

### Pull-down with purified proteins

To verify the in vitro interaction of CSE with AmiC3, pulldown experiments using magnetic MagStrep^®^ “typ3” XT Beads (IBA Lifesciences GmbH) were conducted as described previously (Selim et al, 2023) using purified proteins in the presence or absence of CaCl_2_. AmiC3 was purified as N-terminal His-tagged protein in *pET15b* plasmid as described previously (Zheng et al, 2017) using oligonucleotides (#172 and #165), while CSE was used as C-terminal Strep-tagged. In the pulldown experiments, 20 µM of strep-tagged CSE was pre-incubated on MagStrep “type3” XT beads (IBA GmbH, Göttingen) for 15 min, then for 1 h at 28 °C with 20 µM of His-tagged AmiC in buffer (20 mM Tris-HCL pH 7.9, 100 mM NaCl) with gentle rotation to prevent sedimentation of the beads. To get rid of the excess unbound proteins, the magnetic beads were extensively washed at least six times with 500 µl of buffer. A final washing step with 20 µl was used later as a wash control to make sure that the excess unbound proteins are successfully. Finally, the elution was performed by the addition of 20 μl of either Strep-BXT Biotin Elution Buffer (IBA). The co-elution of AmiC3 with CSE was observed by running all the elution fractions vs. the last wash fraction on a 4–15% SDS Q-PAGE™ TGN Precast Gel (SMOBIO Technology, Inc) at 150 V for 60 min. The proteins were stained using Blauer Jonas (German Research Products Ltd).

### Mass photometry

Mass photometry experiments were conducted according to (Samir et al, 2025; Wallner et al, 2026; Wu and Piszczek, 2021). Clean silicon gaskets (3 mm diameter × 1 mm depth, Grace Bio-Labs, Bend, OR, USA) were cut into nine-well squares and mounted on clean, dry (No. 1.5H) coverslips (24 × 5, Marienfeld, Germany). Prior to measurements, immersion oil was applied to the microscope objective, and the coverslip was fixed in place. Protein samples were diluted in buffer (20 mM Tris-HCl, pH 7.9, 100 mM NaCl) to nanomolar concentrations. Data was acquired with the TwoMP mass photometer (Refeyn, Oxford, UK) using AcquireMP software, following the manufacturer’s guidelines. Each protein was measured individually, followed by a mixture of both proteins in the presence or absence of 2 mM CaCl₂. For focusing, 15 μl of buffer was added to the well, and the autofocus system was used to determine the optimal focal plane. Subsequently, 5 μl of diluted protein solution was pipetted into the same well and mixed thoroughly. Movie acquisition lasted 60 s per sample, producing approximately 60,000 frames. Each measurement was performed in triplicate, and calibration was conducted prior to each experiment. Data analysis and graph generation were carried out using DiscoverMP software.

### Plunge freezing of *Nostoc* filaments

Prior to plunge freezing, *Nostoc* filaments grown on BG11 plates were resuspended in liquid medium to obtain a dense cell suspension. 3.5 μl of this cell suspension was applied to glow-discharged copper EM grids (R2/2, Quantifoil) and automatically blotted from the back for 12–14 s using a Vitrobot plunge-freezing robot (Thermo Fisher), in which one blotting paper was replaced with a Teflon sheet (Weiss et al, 2017). The sample was subsequently vitrified by plunging into liquid ethane-propane (Tivol et al, 2008), and frozen grids were further stored in liquid nitrogen.

### Cryo-focused ion beam milling

Electron-transparent lamellae through plunge-frozen *Nostoc* filaments were prepared using the SerialFIB software package (Klumpe et al, 2021) on a Zeiss Crossbeam 550 FIB-SEM dual-beam instrument (Carl Zeiss Microscopy, Oberkochen). EM grids were clipped into FIB milling autoloader grids (ThermoFisher Scientific, Waltham, MA) and mounted onto a 40° pre-tilted grid holder (Medeiros et al, 2018) (Leica Microsystems GmbH, Vienna, Austria) using a VCM loading station (Leica Microsystems GmbH, Vienna, Austria). Cryo-grid transfer was performed with a VCT500 cryo-transfer system (Leica Microsystems GmbH, Vienna, Austria). Grids were sputter-coated with a ~ 8 nm-thick layer of tungsten using an ACE600 cryo-sputter coater (Leica Microsystems GmbH, Vienna, Austria) and afterward transferred into the dual-beam instrument equipped with a copper-band cooled mechanical cryo-stage (Leica Microsystems GmbH, Vienna, Austria). An organometallic platinum precursor layer was deposited onto each grid via the gas injection system, and the SEM (3–5 kV, 58 pA) was used to identify suitable milling targets. Milling patterns were placed and executed via the SerialFIB software package, aiming for a final lamellae thickness of ~200 nm by applying gradually reducing currents for rough milling (700, 300, and 100 pA) and for polishing (50 pA). Grids with final lamellae were unloaded and stored in liquid nitrogen until cryo-ET imaging.

### Cryo-electron tomography and tomogram reconstruction

Tilt-series of FIB-milled lamellae were collected on a Titan Krios 300 kV FEG transmission electron microscope (Thermo Fisher) equipped with a K3 direct electron detector (Gatan) combined with a BioContinuum imaging filter (slit width 20 eV). All data were acquired using the SerialEM (Mastronarde, 2005, 2003) and PACEtomo (Eisenstein et al, 2023) software packages. Tilt-series were recorded with a dose-symmetric tilt-scheme covering 120° of angular range, with a pixel size of 2.68 Å at the specimen level, at −6 μm defocus, and with a cumulative electron dose of ~140e− Å^−2^. The acquired images were further processed with the IMOD package (Kremer et al, 1996; Mastronarde, 2008). After motion-correction with “alignframes”, the tilt-series were reconstructed into 4× binned tomograms using weighted back-projection. Cryo-tomograms were furthermore filtered using IsoNet (Liu et al, 2022) to improve contrast for visualization. Segmentations were created using Dragonfly (Heebner et al, 2022) and subsequently imported into ChimeraX, Gaussian filtered, and processed with the surface smoothing function. All segmentations were visualized using ChimeraX.

### Statistical analyses

No methods were used to estimate sample sizes, and no blinding was done. Statistical analyses were generally done using GraphPad Prism software. Significance was tested by unpaired Student’s *t* test. The obtained *P* values are indicated in the respective figure legends.

## Supplementary information

Appendix

Peer Review File

Movie EV1

Movie EV3

Movie EV2

Source data Fig. 3

Source data Fig. 4

Source data Fig. 5

Source data Fig. 7

Figure EV4 Source Data

Figure EV5 Source Data

Expanded View Figures

## Data Availability

All data supporting the findings of this study are available within the paper. The NMR data, atomic coordinates, and structure factors have been deposited in the Protein Data Bank, www.wwpdb.org (PDB ID codes: 9QSB). The cryo-electron tomograms shown in the figures were uploaded to the Electron Microscopy Data Bank (EMDB) with accession numbers EMD-52774, EMD-52775, and EMD-52776. The source data of this paper are collected in the following database record: biostudies:S-SCDT-10_1038-S44318-026-00893-y.
