# Supplementary material for: The calcium-binding protein CSE links Ca2+ signaling with cell-cell communication in cyanobacteria
Source: EMBO J. 2026 Aug 13;45(18):6630–54. doi: 10.1038/s44318-026-00893-y (PMC13578307; doi:10.1038/s44318-026-00893-y)
Supplement: Supplementary file 1 [file 44318_2026_893_MOESM12_ESM.pdf]

Expanded View Figures

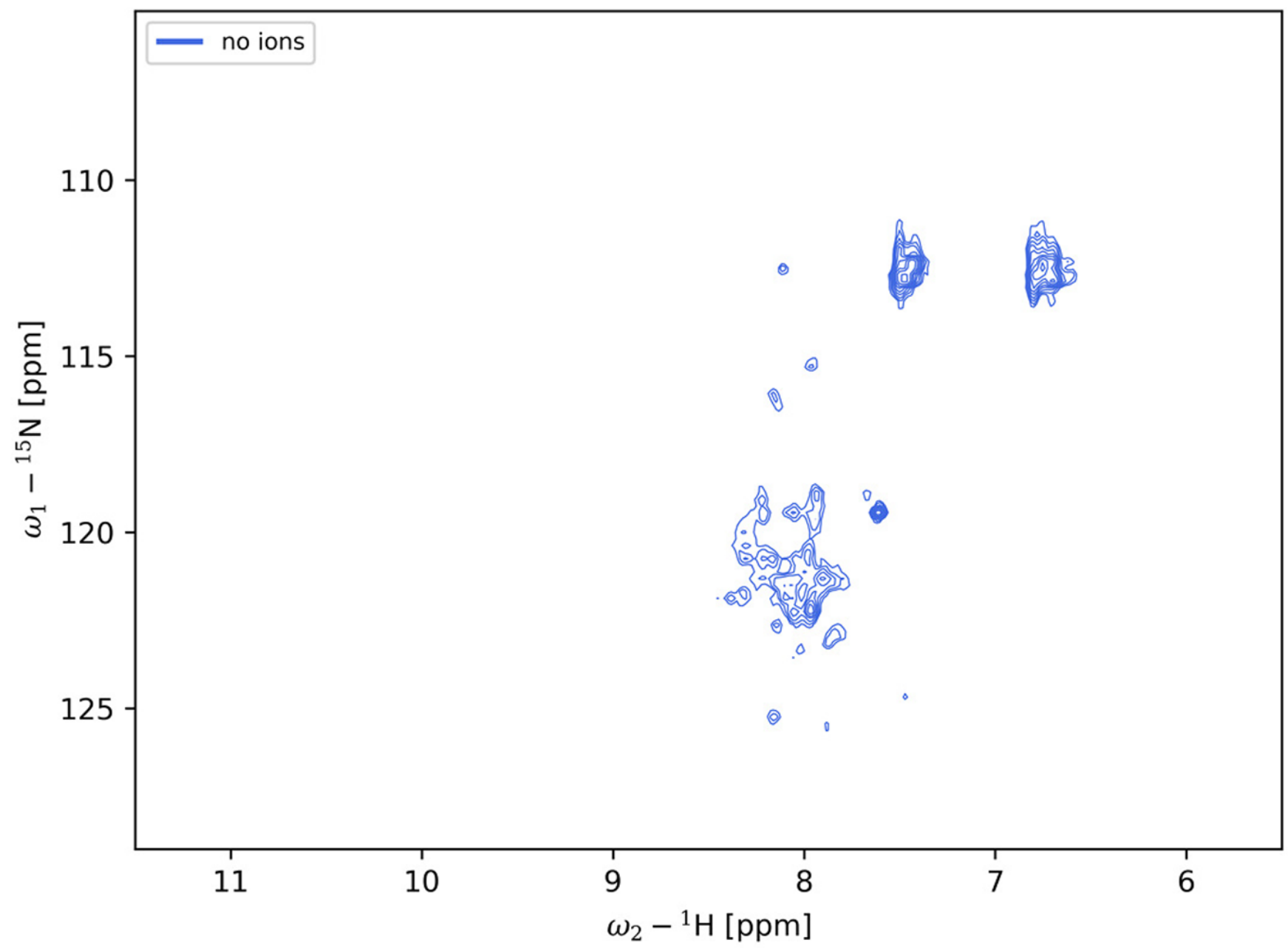

Figure EV1. ^{15}N-HSQC spectrum of the ion-free CSE protein (i.e., without Mg^{2+} or Ca^{2+} ions) from *Nostoc* sp. PCC 7120.

© The Author(s)

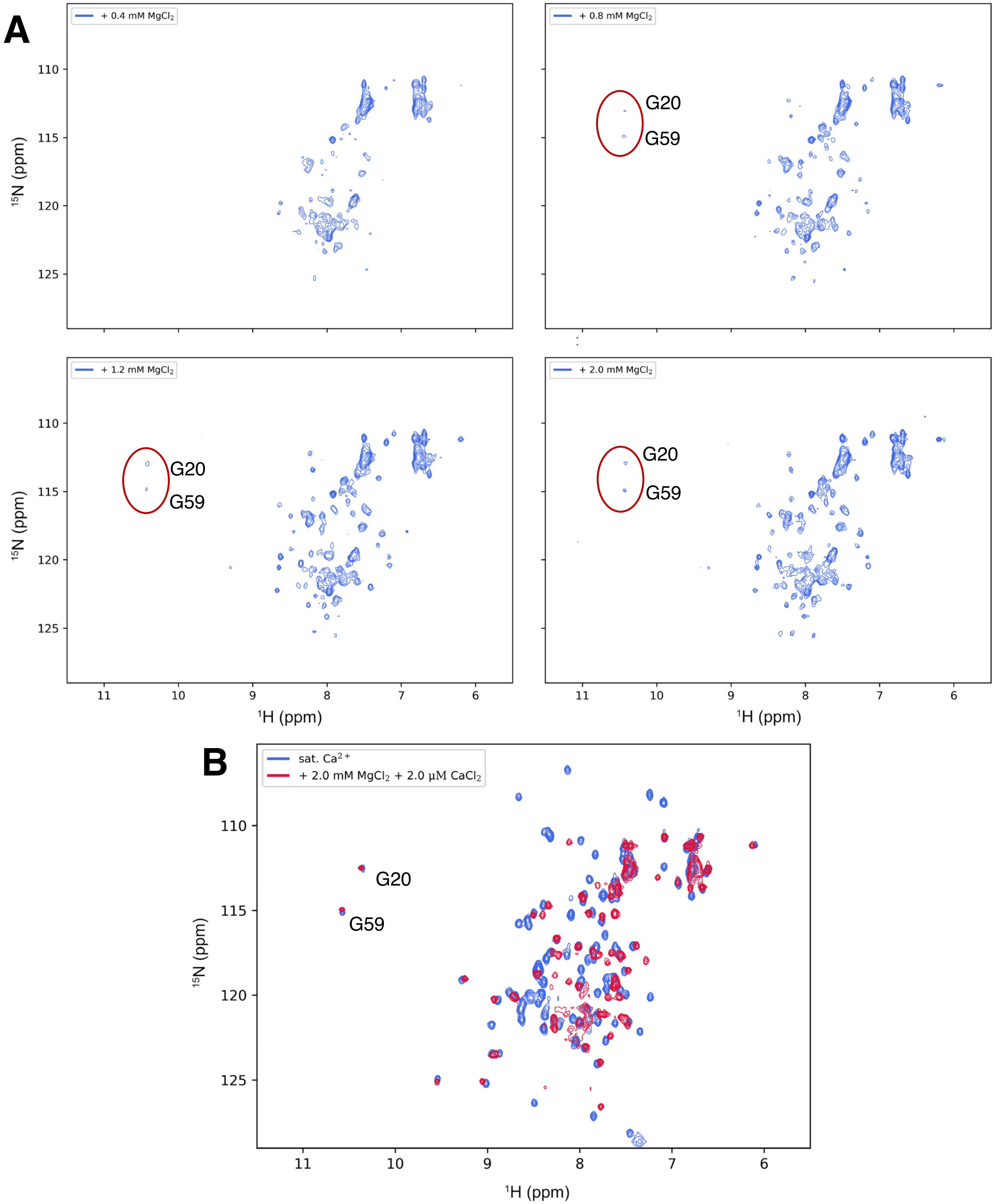
A
+ 0.4 mM MgCl$_2$
+ 0.8 mM MgCl$_2$
+ 1.2 mM MgCl$_2$
+ 2.0 mM MgCl$_2$
G20
G59
^{15}N (ppm)
^{1}H (ppm)
B
sat. Ca^{2+}
+ 2.0 mM MgCl$_2$ + 2.0 μM CaCl$_2$
G20
G59
^{15}N (ppm)
^{1}H (ppm)

Figure EV2. ^{15}N-HSQC spectra of CSE with Mg^{2+} and/or Ca^{2+}.

(**A**) ^{15}N-HSQC spectra of CSE samples titrated with increasing concentrations of $MgCl_2$ (0.4, 0.8, 1.2 and 2.0 mM, as indicated). Peaks corresponding to the characteristic glycine residues in position 6 of the EF-hand loops (G20 and G59) are labeled. (**B**) An overlay of the ^{15}N-HSQC spectra of CSE saturated with Ca^{2+} (blue) and CSE titrated with 2 mM Mg^{2+} (red). The considerable differences in both peak positions and overall dispersion indicate that Ca^{2+} efficiently competes with Mg^{2+} for binding sites.

 © The Author(s)

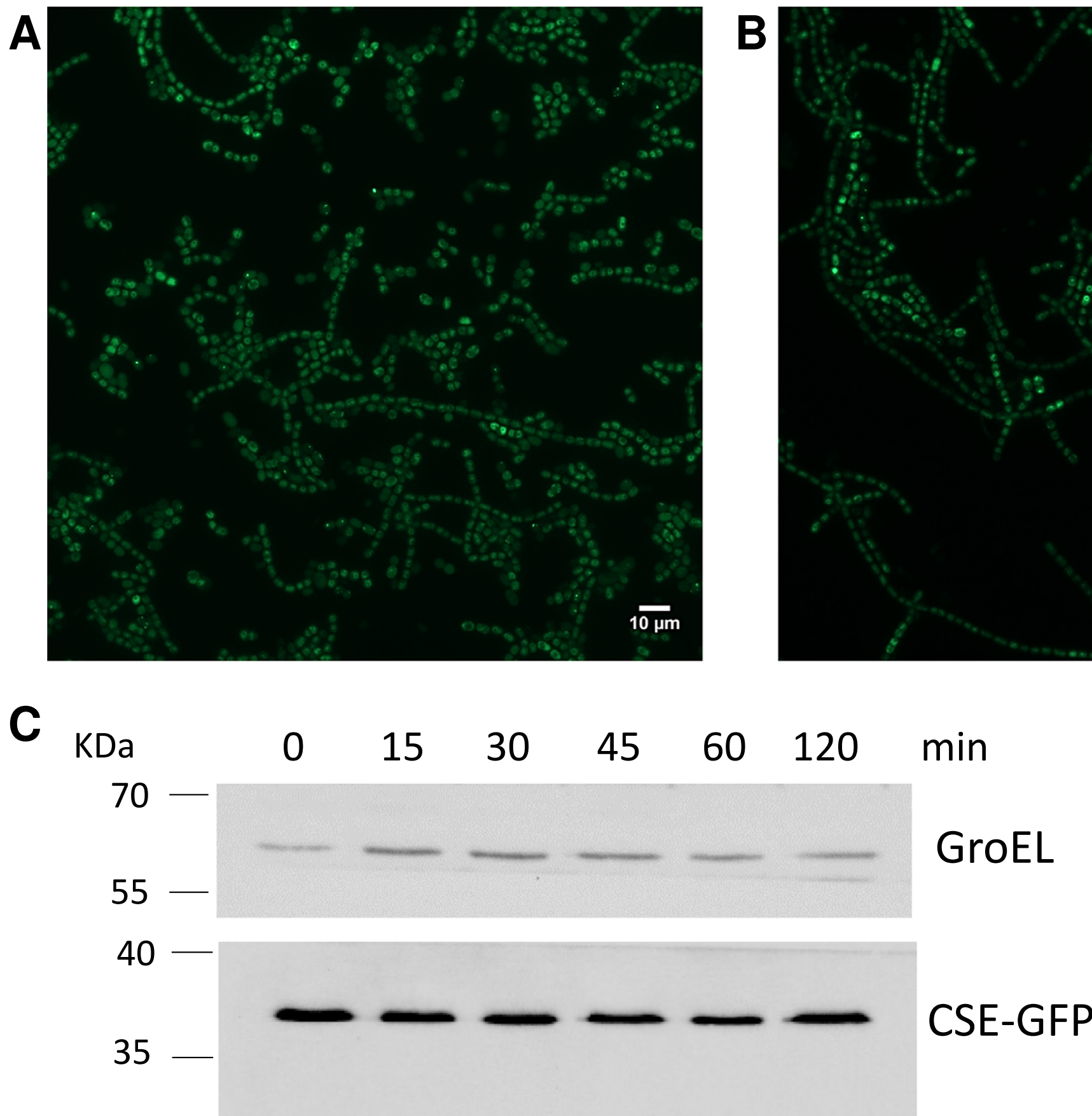

Figure EV3. Cellular localization and abundance of CSE-sfGFP under the control of the native *cse* promoter.

(**A**, **B**) CSE-sfGFP shows cytoplasmic localization, whether *cse-sfgfp* is integrated into the genome (**A**; pMBA226 strain) or expressed from the α-plasmid of *Nostoc* (**B**; pMBA224 strain). Scale bar: 10 µm. Both pMBA226 and pMBA224 are a kind gift from Manuel Brenes-Álvarez (Freiburg University). (**C**) Immunoblot analysis of CSE-sfGFP (≈ 37 kDa) expression using specific α-GFP antibodies under 1% CO_2 and after shifting the cells to 2 h of darkness compared to the expression of GroEL (used as a control marker). The immunoblot analysis revealed that the CSE-sfGFP is abundant in the cell compared to GroEL.

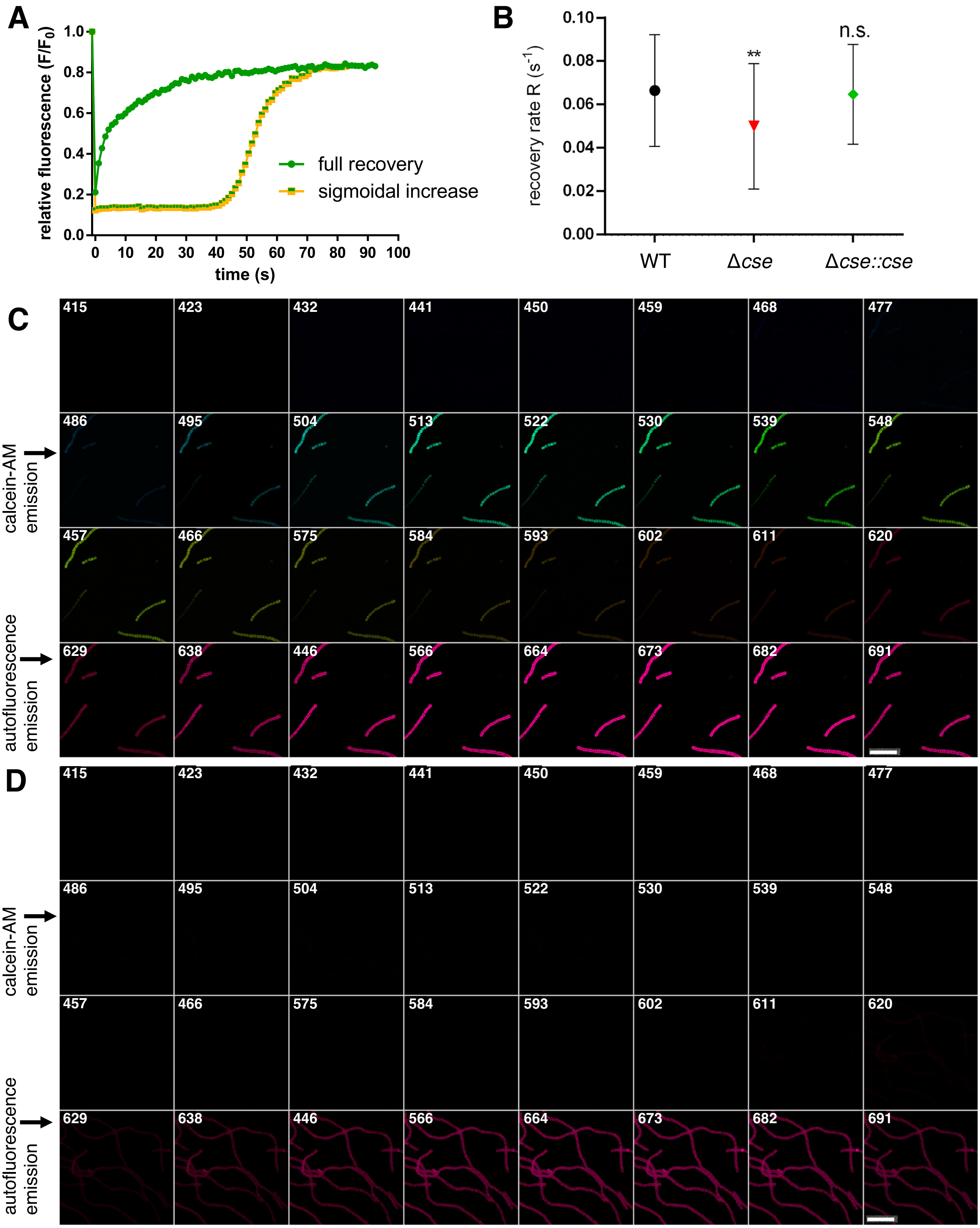
A
relative fluorescence (F/F0)
time (s)
full recovery
sigmoidal increase
B
recovery rate R (s-1)
n.s.
**
WT
Δcse
Δcse::cse
C
D
calcein-AM emission
autofluorescence emission

Figure EV4. Cell–cell communication analysis via FRAP of wild-type (WT) *Nostoc* sp. and its derived *cse* knockout (Δ*cse*) and complementation (Δ*cse::cse*) strains.

(**A**) An example of a sigmoidal recovery curve (in yellow) compared to full recovery response (in green) over time. The sigmoidal increase is characterized by a delay of up to 40–50 s of the fluorescence signal, then rapidly increases, like a full recovery response. First time point is before bleaching (at 1.0 F/F_0) and time point $t = 0$ is measured directly after bleaching (at 0.1–0.2 F/F_0). (**B**) Communication rates calculated from fully recovering cells from FRAP analysis, and showing that the Δ*cse::cse* complementation strain exhibits wild-type (WT) like communication, while Δ*cse* shows a slow communication rate. Significance $^{**}P$ value = 0.006 for Δ*cse* and $P = 0.73$ (not significant) for Δ*cse::cse* complementation strain in Student's *t* test, in comparison to WT. n.s. not significant. Data are pooled from three independent biological replicates with standard deviation (WT, $n = 298$ cells; Δ*cse::cse*, $n = 287$ cells). (**C**, **D**) Lambda scan analysis of *Nostoc* cells excited at 405 nm after staining with (**C**) or without calcein-AM (**D**). Scale bar: 20 µm. Confocal fluorescence micrographs for emission measured in a range from 415 nm to 691 nm (step size of 8.625 nm). Lambda scan analysis revealed no overlap between calcein-AM emission (**C**) and cyanobacterial autofluorescence (**D**). Source data are available online for this figure.

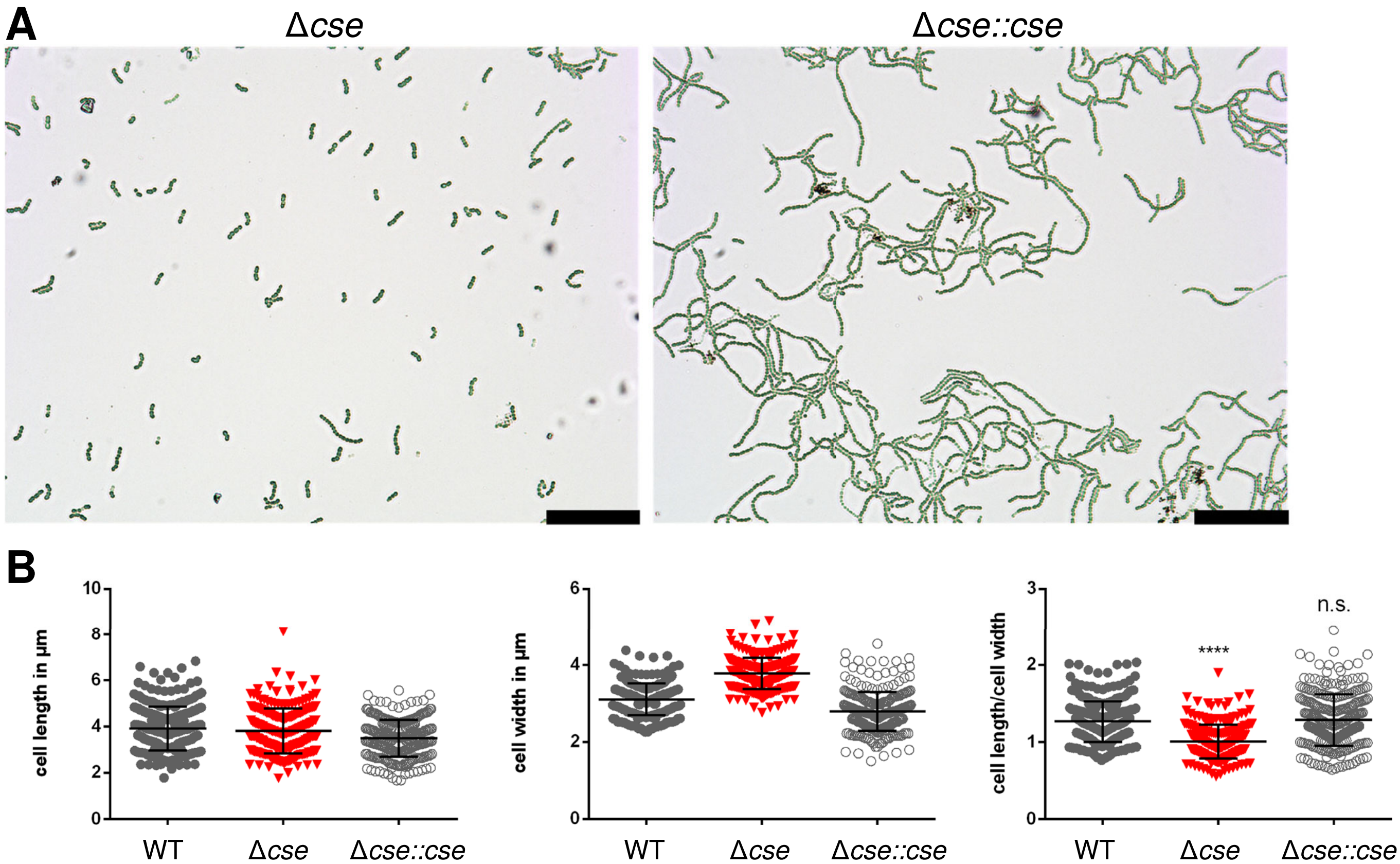

Figure EV5. Ca^{2+}-binding protein CSE controls cell shape and filament integrity.

(**A**) Light microscopy of *cse* knockout (Δ*cse*) and the Δ*cse::cse* complementation strains, showing that the Δ*cse::cse* complementation strain exhibits wild-type (WT) like appearance with regular filaments (as in Fig. 5A) compared to the fragmentation phenotype of the Δ*cse* mutant. Bars, 100 µm. (**B**) Median cell length and cell width of WT and Δ*cse* strains (of Fig. 5B) compared to Δ*cse::cse* complementation strain. The Δ*cse::cse* complementation strain exhibits WT-like appearance compared to the Δ*cse* mutant. Data are pooled from three independent biological replicates (exponentially growing) with standard deviation. Dimensions were measured from multiple filaments across 2–3 images per replicate (WT, $n = 298$ cells; Δ*cse*, $n = 230$ cells; *cse* OE 402 cells). Significance of Δ*cse::cse* strain compared to WT was determined via Student's *t* test: *P* cell length/cell width ratio, $P < 0.0001$ (****) for Δ*cse* strain and $P = 0.47$ (not significant) for Δ*cse::cse* complementation. Whiskers represent standard deviation. n.s. not significant. Source data are available online for this figure.

 © The Author(s)

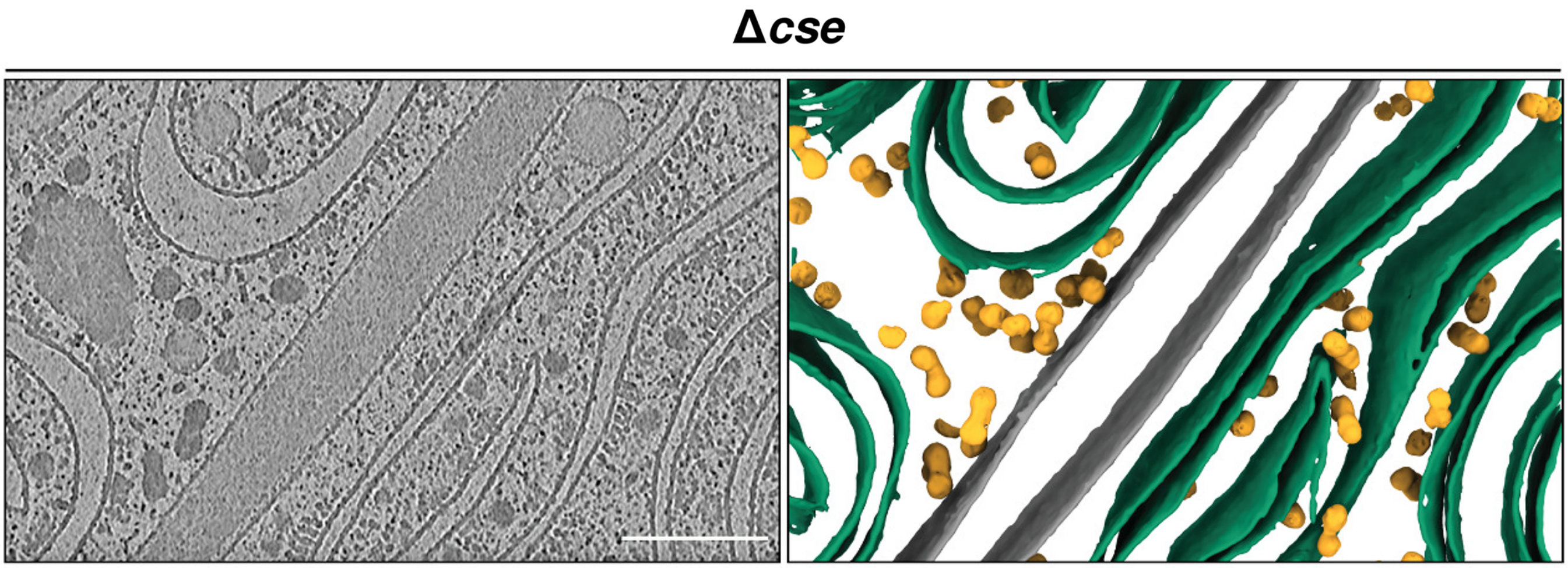

Figure EV6. Cryo-ET reveals a highly reduced number of SJs in the Δ*cse* mutant.

Slice through a cryo-tomogram (shown is a 16-nm-thick slice) of a septum of a *Nostoc* Δ*cse* filament (left), and the corresponding 3D segmentation (right) shows a heavily reduced number of SJs per septum. In this cryo-tomogram of a ~200-nm-thick lamella, no SJs were observed. In comparison, ~8 SJs/tomogram can be observed in WT filaments. Scale bar: 200 nm.

© The Author(s)
