# Supplementary material for: The calcium-binding protein CSE links Ca2+ signaling with cell-cell communication in cyanobacteria
Source: EMBO J. 2026 Aug 13;45(18):6630–54. doi: 10.1038/s44318-026-00893-y (PMC13578307; doi:10.1038/s44318-026-00893-y)
Supplement: Supplementary file 2 [file 44318_2026_893_MOESM1_ESM.pdf]

THE EMBO JOURNAL

Appendix for

The calcium-binding protein CSE links Ca^{2+} signaling with cell-cell communication in cyanobacteria

Authors: Teresa A. Müller[1], Felicia Stahlecker[2], Karolina Roganowicz[3,4], Sherihan Samir[1], Gregor L. Weiss[4], Murray Coles[2], Khaled A. Selim[1,2,5,*]

Affiliations:

[1] Interfaculty Institute of Microbiology and Infection Medicine, Cluster of Excellence "Controlling Microbes to Fight Infections - CMFI", Tübingen University, 72076 Tübingen, Germany

[2] Department of Protein Evolution, Max Planck Institute for Biology, 72076 Tübingen, Germany

[3] Institute of Molecular Biology and Biophysics, Department of Biology, ETH Zürich, Zürich, Switzerland

[4] Institute of Medical Microbiology, University of Zürich, 8006 Zürich, Switzerland

[5] Microbial Biochemistry Group, Institute of Phototrophic Microbiology, Interfaculty Center for Membrane Research – iCMR, Heinrich-Heine University Düsseldorf, Universitätsstraße 1, 40225 Düsseldorf, Germany

*Corresponding authors: **Khaled A. Selim (khaled.selim@uni-duesseldorf.de)**

Table of Contents:

Appendix Figure S1. CSE proteins within cyanobacteria phylum... 2

Appendix Figure S2. CSE-AmiC3 interaction... 3

Appendix Figure S3. Streptavidin magnetic bead-based pull-downs using C-terminal strep-tagged CSE protein (as a bait) in presence of 2 mM Ca^{2+}.. 4

Appendix Figure S4. Genotypic characterization of Δ*cse* knockout mutant. 5

Appendix Table S1. CoMAND ensemble structure statistics of CSE protein, related to Fig. 1. 6

Appendix Table S2. Recovery rates of fully recovering cells as mean values with standard deviation (SD), related to Fig. 3. 7

Appendix Table S3. CSE interactome, showing the list of identified proteins via quantitative MS-based proteomics analysis. 8

Appendix Table S4. Composition of BG 11 media used for *Nostoc* cultivation.............................. 10

Appendix Table S5: Primers used in this study.. 11

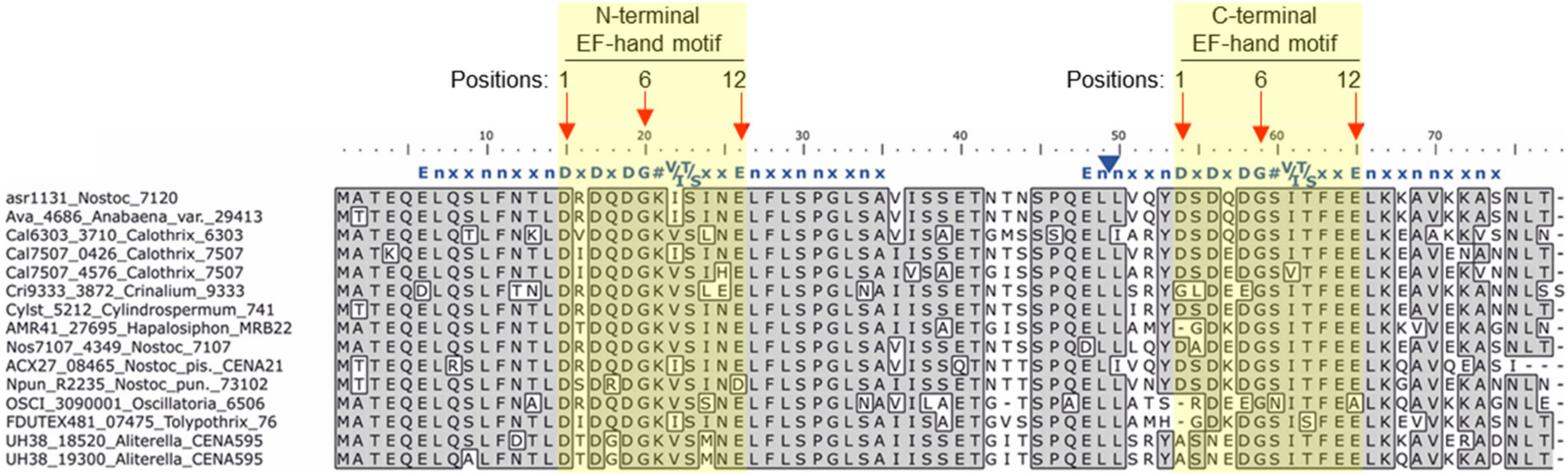

Appendix Figure S1. CSE proteins within cyanobacteria phylum.

Multiple sequence alignment shows conservation of CSE proteins among N_2-fixing multicellular cyanobacteria with 2 characteristic EF-hand Ca^{2+}-binding motifs (positions 1-12; yellow boxes).

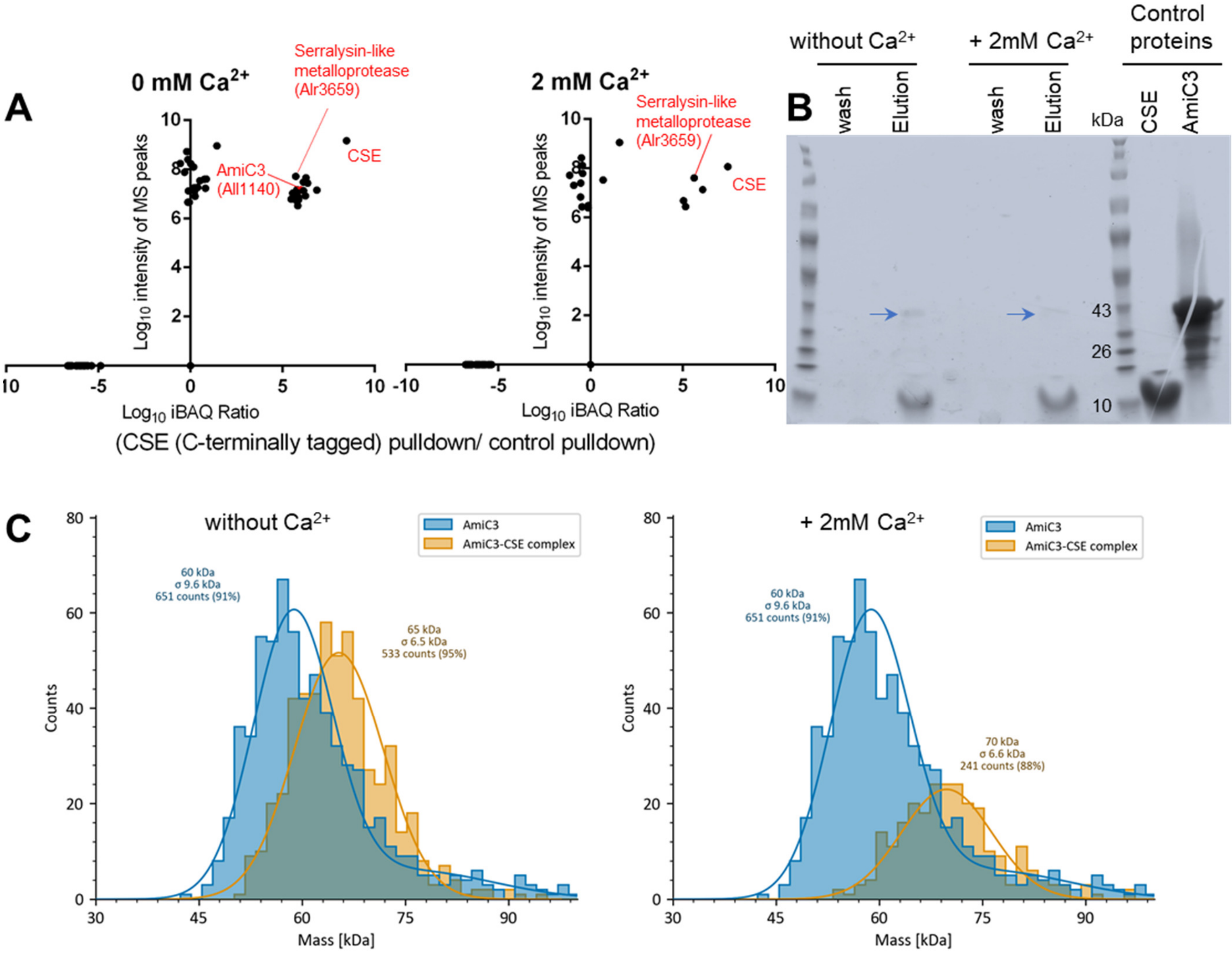

Appendix Figure S2. CSE-AmiC3 interaction.

(A) Streptavidin magnetic bead-based pull-downs using C-terminal strep-tagged CSE protein (as a bait) in the absence or presence of 2mM Ca^{2+}. In absence of Ca^{2+}, both of AmiC3 (All1140) and phosphate binding protein (All2358) were enriched in the CSE pulldown experiment. However, in presence of 2 mM Ca^{2+} CSE-AmiC3 interaction was not detectable. The Serralysin-like metalloprotease (Alr3659) was recognizable under both conditions. Another unrelated strep-tagged PII protein was used as a control pulldown. **(B)** Pulldown analysis of CSE and AmiC3 interaction using purified Strep- and His-tagged proteins, respectively. C-terminal Strep-tagged CSE was immobilized on magnetic streptavidin beads and the coelution of His-tagged AmiC3 (indicated by blue arrow) was checked on SDS-PAGE in presence or absence of 2 mM Ca^{2+}. Presence of 2 mM Ca^{2+} impacted the CSE-AmiC3 interaction. **(C)** Mass photometry analysis of CSE-AmiC3 interaction in presence or absence of 2 mM Ca^{2+}, as indicated and using Strep-tagged CSE and His-tagged AmiC3 proteins. Compared to AmiC3 alone, AmiC3-CSE interaction showed a mass increase (≈ 10 kDa of CSE) corresponding presumably to the complex formation. Again, presence of Ca^{2+} seems to weaken the complex formation as the mass count of AmiC3-CSE interaction is reduced by almost 50% in presence of 2mM Ca^{2+} compared to Ca^{2+}-free complex.

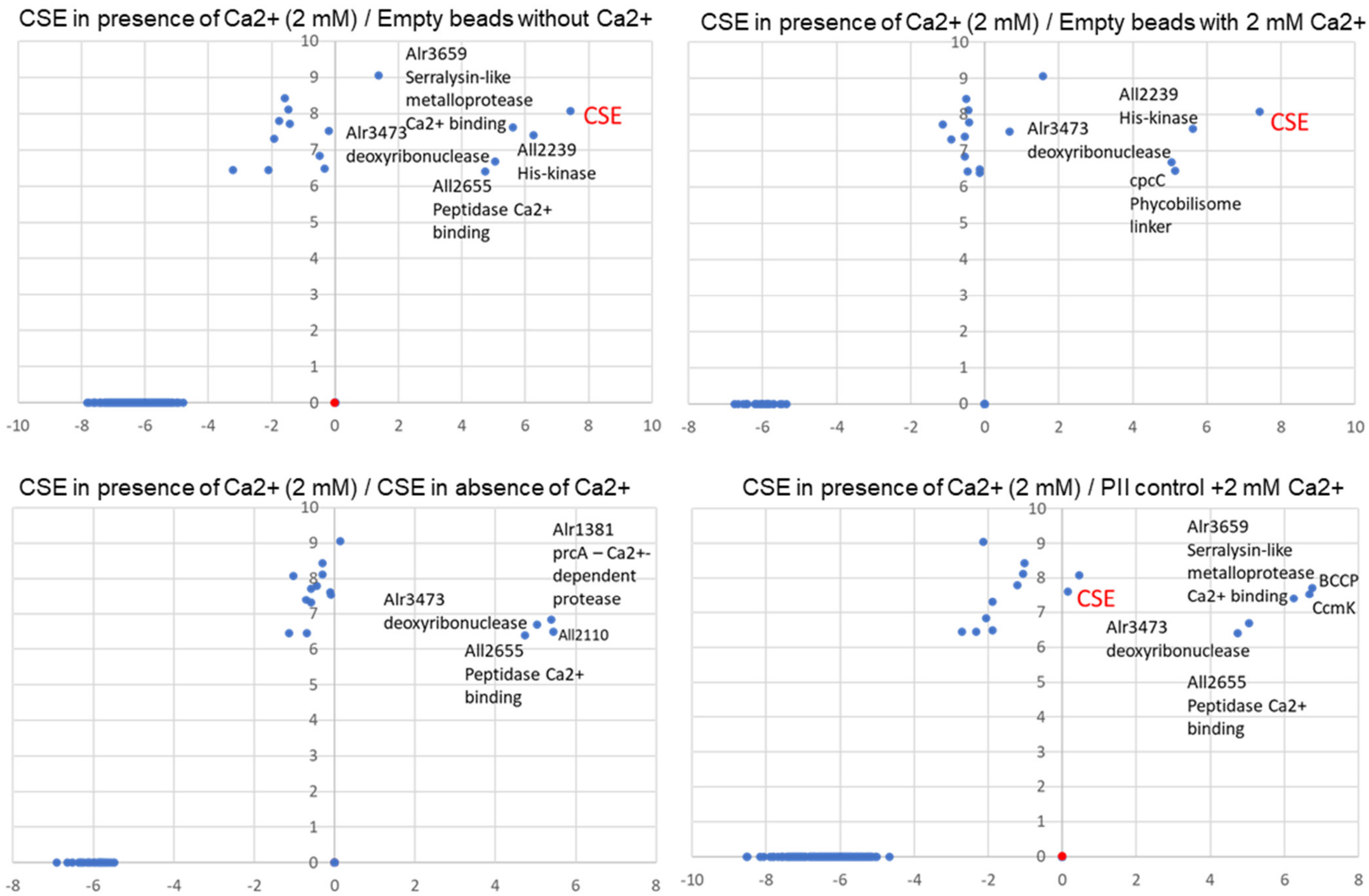

Appendix Figure S3. Streptavidin magnetic bead-based pull-downs using C-terminal strep-tagged CSE protein (as a bait) in presence of 2 mM Ca^{2+} compared to control pulldowns of empty beads (in presence or absence of 2 mM Ca^{2+} buffer) and unrelated PII strep-tagged protein.

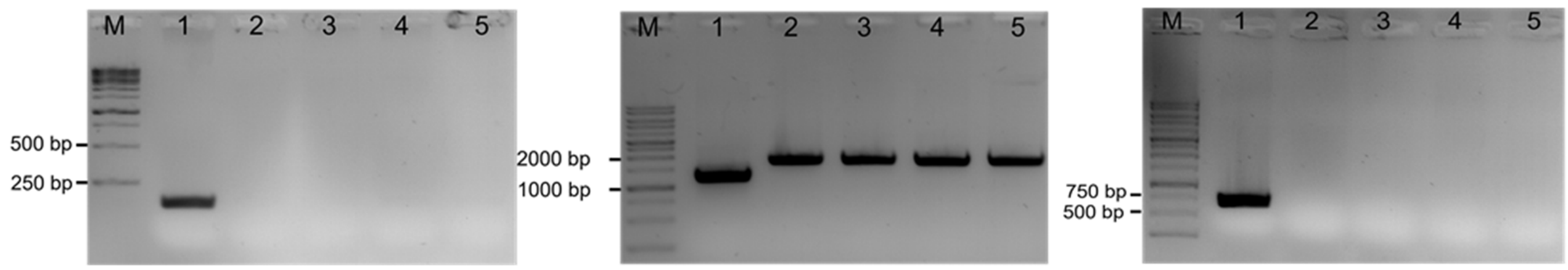

Appendix Figure S4. Genotypic characterization of Δ*cse* knockout mutant.

PCR using different sets of gene-specific primers (see Supplementary Table S5) showing complete segregation of Δ*cse* knockout mutant with insertion of neomycin-resistance-cassette. Lanes: 1 (WT *Nostoc* cells), while 2-5 (Δ*cse* cells).

Appendix Table S1. CoMAND ensemble structure statistics of CSE protein, related to Fig. 1.

**R-Factors**[1]	**CSE**
Coverage[2]	91/92
R_{min}	0.30 ± 0.24
R_{min_bb}	0.20 ± 0.10
R_{diff}	0.10 ± 0.06
R_{mean}	0.31 ± 0.11
**Structure Quality**[3]	
Bonds (Å × 10^{-3})	3.8 ± 0.2
Angles (°)	2.84 ± 0.10
Impropers (°)	3.74 ± 0.35
Ramachandran Map (%)	95.9 / 4.1 / 0.1
Sidechain Regularity (%)	96.9
Clash Score	5.0
**Atomic RMSD (Å)**[4]	
Number of Structures	12
Ordered Residues	2-73
Backbone Heavy Atom	0.77 ± 0.10
All Heavy Atom	1.12 ± 0.12

[1] R-factors averaged across the sequence (± SD) are given for the final ensemble compiled by global optimization: R_{min}, the estimated minimum R-factor for a residue, based on its signal to noise ratio; R_{min_bb}, R_{min} restricted to backbone atoms; R_{diff}, the difference between R and R_{min}; R_{mean}, the average of R-factors used in global optimization. Note that residues contributing to R_{mean} were restricted to those with $R_{min} < 0.6$.

[2] The coverage refers to the number of residues used in R-factor optimization analysis, versus the total number expected from the sequence.

[3] Determined by MOLPROBITY (Chen et al, 2010). The Ramachandran statistic lists the percentage of residues in favored / allowed / disfavored regions of the map (percentiles 98.0 / 99.8 / >99.8). Sidechain regularity lists the percentage in allowed sidechain rotamers (percentile 98.0). The clash score lists steric overlaps > 0.4 Å per 1000 atoms.

[4] The RMSD to the average structure based on superimposition over ordered residues, as defined in the table.

Reference: Chen VB, Arendall WB 3rd, Headd JJ, Keedy DA, Immormino RM, Kapral GJ, Murray LW, Richardson JS, Richardson DC. MolProbity: all-atom structure validation for macromolecular crystallography. Acta Crystallogr D Biol Crystallogr. 2010; 66(Pt 1):12-21. doi: 10.1107/S0907444909042073.

Appendix Table S2. Recovery rates of fully recovering cells as mean values with standard deviation (SD), related to Fig. 3. Recovery rates from FRAP experiments shown in (Fig. 3A) of *Nostoc* sp. PCC 7120 wildtype strain and its derived *cse* knockout (Δ*cse*) and overexpression (*cse* OE) mutants under different calcium concentrations (0 mM, 0.25mM, 1.25 mM), incubated for one or two days (summarized in Fig. 3B).

**[$CaCl_2$]**		**0 mM**			**0.25 mM**			**1.25 mM**		
**Incubated/Strain**		**WT**	***cse* OE**	**Δ*cse***	**WT**	***cse* OE**	**Δ*cse***	**WT**	***cse* OE**	**Δ*cse***
**1 day**	**Mean**	0.0795	0.07097	0.04687	0.08022	0.06942	0.04995	0.08116	0.08052	0.05309
	**SD**	0.02509	0.01645	0.02578	0.03056	0.024	0.02887	0.02368	0.02285	0.02965
**2 days**	**Mean**	0.0908	0.08127	0.06929	0.07779	0.07636	0.05206	0.07356	0.07659	0.04895
	**SD**	0.03281	0.02445	0.03706	0.0241	0.02233	0.0283	0.02418	0.03602	0.02751

Appendix Table S3. CSE interactome, showing the list of identified proteins via quantitative MS-based proteomics analysis. Identified proteins are sorted by their scores, in presence and absence of 2 mM Ca^{2+}.

	Protein ID	Name	gene	function	score	# of peptides	enriched compared to	Ca^{2+} response
CSE pulldown in presence of 2 mM Ca2+	Q06881	BCCP (Biotin carboxyl carrier protein of acetyl-CoA carboxylase)	accB, all5057	Subunit of the acetyl co-A carboxylase complex; common contaminant of streptavidin pulldowns	185.5	5	(PII control)	
	Q8YV71	All2110 protein	all2110	VWFA domain containing; hypothetical protein	54.6	8	(CSE – Ca2+ pulldown)	Yes, identified only in + Ca2+ condition
	Q59149	Calcium-dependent protease	prcA alr1381	Degrades phycobiliproteins in vitro.	48.8	14	(CSE – Ca2+ pulldown)	Yes, identified only in + Ca2+ condition
	Q8YR01	Alr3659 protein	alr3659	Serralysin-like metalloprotease similar to hemolysis-type calcium binding region/ similar to S-layer-RTX protein	48.1	5	(Empty beads – Ca2+ control) and (PII control)	No (identified in both +/- Ca^{2+}
	Q8YYI0;Q8YYI1	Carboxysome shell protein CcmK	ccmK	One of the shell proteins of the carboxysome	5.9	4	(PII control)	
	Q8YTQ8	All2655 protein		Ca2+ binding Peptidase	5.1	1	(CSE – Ca2+ pulldown), (Empty beads + Ca2+ control) and (PII control)	
	Q8YUU2	Histidine kinase	all2239		1.2	1	(Empty beads + Ca2+ control) and (Empty beads - Ca2+ control)	No (identified in both +/- Ca^{2+}
	Q8YRH4; Q8YR34	type I site-specific deoxyribonuclease	alr3473		0.5	1	(CSE – Ca2+ pulldown), (Empty beads + Ca2+ control), (Empty beads - Ca2+ control) and (PII control)	
CSE pulldown in absence of Ca2+	Q06881	BCCP (Biotin carboxyl carrier protein of acetyl-CoA carboxylase)	accB, all5057	Subunit of the acetyl co-A carboxylase complex; common contaminant of streptavidin pulldowns	185.5	5	(PII control)	
	P35796	Phycoerythrocyanin alpha chain	pecA, alr0524	Light-harvesting photosynthetic bile pigment-protein from the phycobiliprotein complex.	161.9	7	(CSE + Ca2+ pulldown)	
	Q8YUI8	Phosphonate ABC transport phosphonate binding component	all2358	belongs to the phosphate/phosphite/phosphonate binding protein family.	54.8	5	(Empty beads + Ca2+ control), (Empty beads + Ca2+ control) and (PII control)	Yes, identified only in – Ca2+ condition
	Q8YR01	Alr3659 protein	alr3659	Serralysin-like metalloprotease similar to hemolysis-type calcium binding region/ similar to S-layer-RTX protein	48.1	5	(Empty beads - Ca2+ control) and (PII control)	No (identified in both +/- Ca^{2+}
	Q8YXS1	All1140 protein AmiC3	all1140	N-acetylmuramoyl-L-alanine amidase	26.9	3	(CSE + Ca2+ pulldown), (Empty beads + Ca2+ control) and (PII control)	Yes, identified only in – Ca2+ condition
	Q8YPD7	All4260 protein	all4260	Hypothetical protein	23.3	3	(PII control)	Yes, identified only in – Ca2+ condition
	Q8YNA5	UPF0234 protein all4662	all4662	cyclic-di-GMP-binding protein	18.5	2	(CSE + Ca2+ pulldown), (Empty beads - Ca2+ control) and (PII control)	Yes, identified only in – Ca2+ condition

	Q8YYS9	All0767	All0767	Ribonuclease	0.5	3	(CSE + Ca2+ pulldown), (Empty beads - Ca2+ control) and (Empty beads + Ca2+ control)	
	Q8YYK9	All0839	All0839	Hypothetical protein; Histone deacetylase family	15.8	2	(CSE + Ca2+ pulldown), (Empty beads + Ca2+ control), (Empty beads - Ca2+ control) and (PII control)	
	Q8Z0I2	Alr0114	Alr0114	Hypothetical protein	10.5	4	(CSE + Ca2+ pulldown) and (Empty beads - Ca2+ control)	
	Q8YQM5	Alr3795	Alr3795	translational initiation	3.4	1	(CSE + Ca2+ pulldown), (Empty beads + Ca2+ control), (Empty beads - Ca2+ control) and (PII control)	
	Q8YU65	Alr2489	Alr2489	Hypothetical protein	1.2	1	(CSE + Ca2+ pulldown), (Empty beads + Ca2+ control), (Empty beads - Ca2+ control) and (PII control)	
	Q8YV65	All2116	All2116	Hypothetical protein	9.5	2	(CSE + Ca2+ pulldown), (Empty beads + Ca2+ control), (Empty beads - Ca2+ control) and (PII control)	
	Q8YUU2	Histidine kinase	all2239		1.2	1	(Empty beads + Ca2+ control) and (Empty beads - Ca2+ control)	No (identified in both +/- Ca^{2+}
	Q8YRE9	All3499	All3499	Hypothetical protein	0.4	1	(CSE + Ca2+ pulldown), (Empty beads - Ca2+ control) and (PII control)	
	Q8YKQ0	Alr7243	Alr7243	similar to ankyrin	5.7	2	(CSE + Ca2+ pulldown), (Empty beads + Ca2+ control), (Empty beads - Ca2+ control) and (PII control)	
	Q8YNW3	Alr4448	Alr4448	Beta-xylanase	2.5	1	(CSE + Ca2+ pulldown), (Empty beads + Ca2+ control), (Empty beads - Ca2+ control) and (PII control)	
	Q8YKH2	Alr7326	Alr7326	Hypothetical protein	3.8	1	(CSE + Ca2+ pulldown), (Empty beads + Ca2+ control), (Empty beads - Ca2+ control) and (PII control)	
	Q8Z099	Alr0199	Alr0199	Hypothetical protein	5.7	3	(CSE + Ca2+ pulldown) and (PII control)	

Appendix Table S4. Composition of BG 11 media used for *Nostoc* cultivation.

component	µM	component	µM
$MgSO_4$ x $7H_20$	304	H_3Bo_3	46.3
$CaCl_2$ x $2H_2O$	245	$MnCl_2$ x 4 H_2O	9.2
K2HPO4	175	$ZnSO_4$ x 7 H_2O	0.77
Na_2CO_3	180	Na_2MoO_4 x 2 H_2O	1.6
$C_6H_8O_7$	31	$CuSO_4$ x 5 H_2O	0.32
$FeC6H_5O_7$	24	CoCl2 x 6 H_2O	0,2
Na_2EDTA x 2 H_2O	3.4	NaNO3	17.6 x10^3

Appendix Table S5. Primers used in this study. Different oligonucleotides, which bind inside or up- and downstream of *cse* gene

Purpose	Oligo name	Oligo #	Sequence	Product length	
				WT	Δ*cse*
To prove segregation of Δ*cse* mutant	Internal forward	#70	ACCGAGCAAGAGCTTCAATC	173 bp	No product
	Internal reversed	#71	CTACCGTCTTGGTCTGAATC		
	External forward	#122	ATTTGTCCCATCCGACTCAG	1231 bp	1713 bp
	External reversed	#124	ATTGCCCACCATAACCCAAC		
	Internal forward 2	#81	GCCCTGGTTTAAGTGCAGTC	712 bp (with #124)	No product
For overexpression of CSE proteins	Forward WT CSE (C-terminal Strep-tagged)	#119	TAGAAATAATTTTGTTTAACTTTAAGAAGGAGATATACAAatggcaaccgagcaagagct	For expression of C-terminal Strep-tag II wildtype CSE protein	
	Reversed WT CSE (C-terminal Strep-tagged)	#120	GGTCTTATTTTTCGAACTGCGGGTGGCTCCAGCTAGCCATggttaaattacttgctttcttaactgc		
	Forward WT CSE (N-terminal Strep-tagged)	#115	CTGGAGCCACCCGCAGTTCGAAAAAGGCGCCGAGACCGCGatggcaaccgagcaagagct	For expression of N-terminal Strep-tag II wildtype CSE protein	
	Reversed WT CSE (N-terminal Strep-tagged)	#116	CAAGCTTAGTTAGATATCAGAGACCATGGTCCCCCTGCctaggttaaattacttgctttcttaactgc		
	Forward D54/56/58A	#148	AGTACAGTATGCTTCAGCCCAAGCCGGTAGTATTACC	For expression of N-terminal Strep-tag II CSE (D54/56/58A) protein variant	
	Reversed D54/56/58A	#149	AGCAACTCTTGGGGGCTATTGGTATTC		
AmiC3 overexpression	#TM172_pTM30_amiC3fw2	#172	CAGCAGCGGCCTGGTGCCGCGCGGCAGCCATATGCTcgagatgagatatggaattgatattggtc	For expression of N-terminal His-tagged AmiC3 protein	
	#TM165_pTM30_amiC3rv	#165	CCAACTCAGCTTCCTTTCGGGCTTTGTTAGCAGCCGGATCctaaccgattagtctttgcc		
	pET15b linear fw	#223	GATCCGGCTGCTAACAAAGC	Linearization of pET15b plasmid	
	pET15b linear rev	#224	CTCGAGCATATGGCTGCCGC		
