# Supplementary material for: The calcium-binding protein CSE links Ca2+ signaling with cell-cell communication in cyanobacteria
Source: EMBO J. 2026 Aug 13;45(18):6630–54. doi: 10.1038/s44318-026-00893-y (PMC13578307; doi:10.1038/s44318-026-00893-y)
Supplement: Supplementary file 3 [file 44318_2026_893_MOESM2_ESM.pdf]

THE EMBO JOURNAL

The calcium-binding protein CSE links Ca2+ signaling with cell-cell communication in cyanobacteria

Teresa Müller, Felicia Stahlecker, Karolina Roganowicz, Sherihan Samir, Gregor Weiss, Murray Coles, and Khaled Selim

Corresponding authors: Khaled Selim (Khaled.Selim@uni-duesseldorf.de)

Review Timeline:

Submission Date:	11th May 26
Editorial Decision:	12th Jun 26
Revision Received:	25th Jun 26
Editorial Decision:	17th Jul 26
Revision Received:	21st Jul 26
Accepted:	28th Jul 26

Editor: Ieva Gailite

Transaction Report:

Please note that the manuscript was previously reviewed at another journal. The manuscript was assessed by an arbitrator based on the reports from that previous external submission.

(Note: With the exception of the correction of typographical or spelling errors that could be a source of ambiguity, letters and reports are not edited. Depending on transfer agreements, referee reports obtained elsewhere may or may not be included in this compilation. Referee reports are anonymous unless the Referee chooses to sign their reports.)

Response to reviewers

Round: #2

Reviewer 1:

The authors have made a good response to several of the issues raised on the first submission. The analysis of the cell communication phenotype of the CSE mutant is excellent. Unfortunately, I don't think the revised manuscript really addresses the key point raised by both reviewers - does CSE act as a specific calcium sensing signal transducer or is just a calcium buffer, with the various phenotypic effects arising because of perturbation of calcium homeostasis? There are hints that the latter scenario may be the case:

a. Perturbation of CSE leads to a really diverse set of phenotypic effects: disruption of photosynthesis (previously reported), changes in cell dimensions, perturbation of intercellular communication. It doesn't exclude that CSE may be directly involved in multiple signaling pathways, but it would be simpler to assume that all these effects arise as consequence of perturbed levels of free Ca2+ in the cytoplasm.

b. The authors' new data with CSE-sfGFP (Fig. S3) suggest that CSE is dispersed in the cytoplasm and extremely abundant (considerably more so even than GroEL). Both those things tend to suggest a calcium buffer rather than a direct signal transducer.

- First, we would like to thank the reviewer #1 for his/her engagement in our work and the detailed suggestions to improve the manuscript.
- We agree with the reviewer that the main reason for the various phenotypes associated with CSE arises from the perturbation of **Ca^{2+}-homeostasis**. However, we wish to clarify a potential misunderstanding regarding our use of the term 'signaling'. In this context, we were referring to the overall Ca^{2+}-signaling pathway, in which CSE acts as a Ca^{2+}-buffer protein, not as a signal transducer. Our NMR (Fig. 1) and **ITC (new Figure 2)** experiments clearly indicated that CSE acts as a Ca^{2+}-buffer protein similar to other EF-hand containing proteins involved in the control of Ca^{2+}-homeostasis, while it becomes totally unfolded in absence of divalent cations. This finding is consistent with CSE being abundant in the cell **(new Figure S3).** Therefore, 'signaling' was used in reference to Ca^{2+} as a second messenger, but now revised through the manuscript.
- Moreover, we stated clearly in several places that CSE acts mainly as Ca^{2+}-buffer protein to control Ca^{2+}-homeostasis (e.g. L240-242; L273; L339-400). Also, we deleted or corrected the “signaling” term in several places to Ca^{2+}-homeostasis for better clarity. We changed the title to ***“The Ca^{2+}-binding protein CSE links Ca^{2+}-homeostasis with cell-cell communication in multicellular cyanobacteria”***

Response to reviewers

Figure for reviewers and associated text removed.

- So, there is certainly a question to answer concerning the reasons behind the phenotypic effects in the CSE knockout. The authors have provided new data from pull-downs that suggest a direct interaction between CSE and AmiC3 (Fig S7), however these data are presented in rather a strange way: they are not mentioned at all in Results but first introduced in the Discussion, where the authors acknowledge that they are preliminary (line 396). I think that if the authors wish to draw conclusions from these data they should present them fully, for example providing the full ranked list of the "other targets" that were detected in the CSE pulldowns (line 396).

This is indeed a very interesting point. the function as a Ca^{2+}-buffer protein does not exclude the possibility that CSE is also directly involved in multiple signaling pathways, similar to the EF-hand proteins family (e.g. calmodulin). To investigate this, the pulldown experiments are now properly included in the results section, along with the full ranked list of identified proteins (**new Table S3**).

Next, we carefully checked these pulldowns (conducted in the presence or absence of Ca2+ to define the Ca2+ -response) and included 3 independent controls (an unrelated protein and 2 empty-bead controls challenged with Nostoc extract in the presence or absence of Ca2+) to exclude non-specific proteins and to focus on proteins that respond to different Ca2+ concentrations (**new Figure S7**). Finally, we applied a strict cut-off to sort for promising CSE-targets, requiring a minimum score of 20 and enrichment in CSE-based pulldowns compared to at least two controls (**new Figure S8**). This analysis identified three potential CSE-targets, two of which were identified in absence of Ca2+ (phosphate binding protein All2358 and amidase AmiC3 All1140), suggesting that Ca2+-binding to CSE disrupts the complexes formation, while the third target (Ca^{2+}-dependent Serralysin-like metalloprotease Alr3659) was enriched regardless of Ca2+ presence. Of these targets, AmiC3 appeared as a promising target due to its involvement in nanopores formation and controlling the diameter of nanopores. We further validated the interaction of CSE with AmiC3 with 2 independent biophysical methods of pulldown assays and mass photometry (**this new data in now in new Figure S7**). This part is now properly discussed (L397-400 and L425-432).

Other specific points (in the order in which they occur in the text).

1. line 52: "between organisms, deviated billion years ago". The whole sentence is very mangled English. Much better would be "...conserved in organisms that diverged billions of years ago" >> Changed

2. line 271: "the absence of CSE" >> Changed

3. Lines 274-275 "supporting a crucial role for Ca2+ signaling via CSE...". Here, and in many subsequent places in the text (eg lines 288, 341, 355, 367) the authors seem to jump to the conclusion that CSE is a signal transducer, without ever providing convincing evidence that it is not simply a calcium buffer. See comments above. The question is never discussed clearly in the text. The results do strongly indicate that Ca2+ levels are important for regulating cell communication (amongst other things) but to me it isn't at all clear that CSE is directly involved in signaling. >> Modified to be focused more on homeostasis

4. Line 328. Rather pedantic, but it should be "fewer" not "less". >> Changed

5. Line 346. "Filamentous cyanobacteria are one of the oldest multicellular organisms on Earth". It suggests that all filamentous cyanobacteria are the same, which they aren't. They are very diverse. Better "...are among the oldest multicellular organisms..." >> Changed

6. Line 384. "...the deletion of the Ca2+-binding protein CSE is very important to form a proper septal disc...". The authors surely mean "...the Ca2+-binding protein CSE is required for correct formation of the septal discs.."! >> Changed

Response to reviewers

Reviewer: 2

The authors have appropriately addressed the main concerns and requested changes, and their replies to the other reviewer questions also help clarify some of the concerns raised in my comments. The manuscript has improved in clarity and reduced ambiguity. However, I still have two minor comments regarding the sigmoidal-type recovery curve and the concept of 'full recovery'.

First, we would like to thank the reviewer #2 for his/her kind words and the overall enthusiasm about our work.

1. Now seeing the sigmoidal curves, I believe one question arises. To my knowledge (and if I am wrong, please include reference to this) this is the first time a sigmoidal recovery pattern is explicitly described in the context of FRAP experiments in cyanobacteria (since you report it in WT cells, I believe not necessarily the first time it has been noticed). Have the authors checked by Lambda scan if the emission peak of the recovered bleached cell of the sigmoidal cases matches to the emission peak as the one being recovered in bleached cells from "full recovery" examples? This could discern if this is actual Calcein peak or if it is an increased autofluorescence entering in the wavelength range of Calcein detection. Nonetheless, if this type of recovery is mentioned, authors should briefly acknowledge that this is the first time such a pattern is reported in cyanobacteria FRAP experiments and that there is currently no clear evidence on what may cause this observed delayed fluorescence recovery. Thanks for this comment!

We now acknowledged that this type of sigmoidal fluorescence recovery has not been previously reported in cyanobacteria FRAP studies, for which the reasons are not yet clear (L255-258). Moreover, to exclude any possible overlap between calcein-AM emission and autofluorescence of bleached cells and to make sure that sigmoidal increase corresponds to calcein-AM emission, a lambda scan was recorded using a Zeiss LSM 880 confocal microscope. *Nostoc* cells (unstained or stained with calcein-AM) were excited with a wavelength of 405 nm and emission was captured in 32 pictures from 415 nm to 691 nm (step size of 8.625 nm), which were assembled using the Zeiss zen blue software. Lambda scan analysis revealed no overlap between calcein-AM emission and cyanobacterial autofluorescence **(new Fig. S4C,D)**, supporting that the fluorescence recovery in FRAP experiments arises specifically from calcein-AM recovery.

2. In the new Fig. S4 it can be seen the so-called 'full recovery' curve where recovery reaches approximately 80%, so why call it full recovery?, and also this raises the question: why is the sigmoidal curve not also considered 'full recovery' if it reaches a similar recovery percentage at the end? I would suggest reconsidering the terminology used.

The critical distinction here is the shape of the recovery curve (the kinetics "rate"), not the final extent of recovery. While a final recovery of ~80% of the initial signal is typical for such analyses (Key Ref. here: Fig. 2B; https://www.cell.com/cell/fulltext/S0092-8674(19)30626-9), the striking feature of the sigmoidal curve is the initial lag phase—a delay in the calcein-AM signal increase. Thus, the pattern of the sigmoidal curve distinguishes it from a simple exponential "full" recovery, where the signal increases immediately.

1st Editorial Decision **12th Jun 2026**

Dear Khaled,

Thank you for submitting your revised manuscript to The EMBO Journal. I sincerely apologise for the delay in communicating the decision due to conference travel. I have now received input from an arbitrating advisor, who finds that the reviewers' concerns from the previous assessment round have been sufficiently addressed. I will therefore be happy to accept the manuscript for publication in The EMBO Journal after its reformatting along the guidelines included below and in the attached document.

Please feel free to contact me if you have any further questions regarding this final revision. You can use the link below to upload the revised files.

Thank you for the opportunity to consider your work for publication, and I look forward to receiving the reformatted version.

With best wishes,

Ieva

Ieva Gailite, PhD
Senior Scientific Editor
The EMBO Journal
Meyerhofstrasse 1
D-69117 Heidelberg
Tel: +4962218891309
i.gailite@embojournal.org

Instructions for preparing your revised manuscript:

Read our guidance for manuscript revisions and related editorial policies: https://link.springer.com/journal/44318/submission-guidelines#cms-Revised-submissions

Please also check that the title and abstract of the manuscript are brief, yet explicit, even to non-specialists.

When assembling figures, please refer to our figure preparation guideline in order to ensure proper formatting and readability in print as well as on screen:
https://media.springernature.com/original/springer-cms/rest/v1/content/27825798/data/v1

IMPORTANT: When you send the revision we will require
- a point-by-point response to the referees' comments, with a detailed description of the changes made (as a word file).
- a word file of the manuscript text.
- individual production quality figure files (one file per figure)
- a complete author checklist
- Expanded View files (replacing Supplementary Information)
- a Reagents and Tools Table as part of the Methods section

Please remember: Digital image enhancement is acceptable practice, as long as it accurately represents the original data and conforms to community standards. If a figure has been subjected to significant electronic manipulation, this must be noted in the figure legend or in the 'Methods' section. The editors reserve the right to request original versions of figures and the original images that were used to assemble the figure.

We realize that it is difficult to revise to a specific deadline. In the interest of protecting the conceptual advance provided by the work, we recommend a revision within 3 months (10th Sep 2026). Please discuss the revision progress ahead of this time with the editor if you require more time to complete the revisions.

Referee #1:

I think the authors have made a good response to the reviewers' comments from the previous rounds. I have no further modifications to suggest.

The authors addressed the editorial issues.

Dear Khaled,

Thank you for submitting a reformatted version of your manuscript. I sincerely apologise for the delay in the processing of your revised manuscript due to conference travel and the resulting backlog.

I have now gone through the revised version, and I am afraid that there remain a few formatting aspects as outlined below that still need to be implemented in the manuscript before its acceptance:

1. Please make sure that the order of the sections in the manuscript is as follows: Abstract / Introduction / Results / Discussion / Methods / Data Availability / Acknowledgements / Disclosure and Competing Interests Statement / References / Figure Legends / Tables / Expanded View Figure Legends.
2. Please fill in rows 57-59 in the Author Checklist.
3. Please merge "Funding" section with "Acknowledgements" in the manuscript text file.
4. Please make sure that the funding information is correct and identical both in the manuscript and our online system, as the information from our submission system will be linked to the article in PubMed.
5. CRediT has replaced the traditional author contributions section because it offers a systematic, machine-readable author contributions format that allows for more effective research assessment. Please remove the Authors Contributions from the manuscript and use the free text boxes beneath each contributing author's name in our online submission system to add specific details on the author's contribution. More information is available in our guide to authors.
6. Please rename the videos into Movie EV1-EV3 and update the callouts and the legends accordingly. Please remove their legends from the manuscript text file and zip with each movie file as a simple README or text file.
7. Please remove the Reagents and Tools table from the manuscript text file, leaving it only as the separately uploaded file. Please remove synopsis text from the file.
8. In the Reagents and Tools table, please correct the callout of Tables S4- S5 to Appendix Tables S4- S5.
9. Figure panels 2A,B; EV3A-C; EV5A-B are not mentioned in the manuscript text; please add the corresponding callouts.
10. In the "Data Availability" section, please add resolvable links for datasets 9QSB, EMD-52776, EMD-52774.
11. In the Appendix, please add page numbers to the Table of contents.
12. Our data editors have flagged the following issues in figure legends that need correcting:
- Please provide the exact p values in the legends of figures 3B, 5B, 7C-E; EV4 B, EV5 B.
- Please define the box plots in terms of minima, maxima, centre, bounds of box and whiskers, and percentile in the legends of figures 7E.
- Please describe the number and nature of replicates in the legends of figures 5B, EV4 B, EV5 B.
- Please define the error bars in the legend of figure EV4 B.
- Please note that scale bar and its definition are missing for figures EV3 A, B; EV4 C, D.

Finally, we would like to propose textual edits in the manuscript title, abstract and synopsis to improve the accessibility to our more general readership. We have also selected a short blurb that will accompany the title of the manuscript in our online table of contents. Please take a look at the proposed text changes in the attached file and let me know if any corrections are needed.

Please feel free to contact me if have any questions regarding this final revision. Thank you again for giving us the chance to consider your manuscript for The EMBO Journal. I look forward to receiving the final version.

With best regards,

Ieva

Ieva Gailite, PhD
Senior Scientific Editor
The EMBO Journal
Meyerhofstrasse 1
69117 Heidelberg
i.gailite@embojournal.org

We realize that it is difficult to revise to a specific deadline. In the interest of protecting the conceptual advance provided by the work, we recommend a revision within 3 months (15th Oct 2026). Please discuss the revision progress ahead of this time with the editor if you require more time to complete the revisions.

The authors addressed the remaining formatting issues.

Editor accepted the manuscript.

EMBO Press Author Checklist

Corresponding Author Name: Khaled Selim	
Journal Submitted to: The EMBO Journal	
Manuscript Number: EMBOJ-2026-124599-T	

USEFUL LINKS FOR COMPLETING THIS FORM
The EMBO Journal - Author Guidelines
EMBO Reports - Author Guidelines
Molecular Systems Biology - Author Guidelines
EMBO Molecular Medicine - Author Guidelines

Reporting Checklist for Life Science Articles (updated January
This checklist is adapted from Materials Design Analysis Reporting (MDAR) Checklist for Authors. MDAR establishes a minimum set of requirements in transparent reporting in the life sciences (see Statement of Task: 10.31222/osf.io/9sm4x). Please follow the journal's guidelines in preparing your
Please note that a copy of this checklist will be published alongside your article.

Abridged guidelines for figures
1. Data
The data shown in figures should satisfy the following conditions:
- the data were obtained and processed according to the field's best practice and are presented to reflect the results of the experiments in an accurate and unbiased manner.
- ideally, figure panels should include only measurements that are directly comparable to each other and obtained with the same assay.
- plots include clearly labeled error bars for independent experiments and sample sizes. Unless justified, error bars should not be shown for technical
- if n<5, the individual data points from each experiment should be plotted. Any statistical test employed should be justified.
- Source Data should be included to report the data underlying figures according to the guidelines set out in the authorship guidelines on Data

2. Captions
Each figure caption should contain the following information, for each panel where they are relevant:
- a specification of the experimental system investigated (eg cell line, species name).
- the assay(s) and method(s) used to carry out the reported observations and measurements.
- an explicit mention of the biological and chemical entity(ies) that are being measured.
- an explicit mention of the biological and chemical entity(ies) that are altered/varied/perturbed in a controlled manner.
- the exact sample size (n) for each experimental group/condition, given as a number, not a range;
- a description of the sample collection allowing the reader to understand whether the samples represent technical or biological replicates (including how many animals, litters, cultures, etc.).
- a statement of how many times the experiment shown was independently replicated in the laboratory.
- definitions of statistical methods and measures:
  - common tests, such as t-test (please specify whether paired vs. unpaired), simple χ2 tests, Wilcoxon and Mann-Whitney tests, can be unambiguously identified by name only, but more complex techniques should be described in the methods section;
  - are tests one-sided or two-sided?
  - are there adjustments for multiple comparisons?
  - exact statistical test results, e.g., P values = x but not P values < x;
  - definition of 'center values' as median or average;
  - definition of error bars as s.d. or s.e.m.

Please complete ALL of the questions below.
Select "Not Applicable" only when the requested information is not relevant for your study.

Materials

Newly Created Materials	Information included in the manuscript?	In which section is the information available? (Reagents and Tools Table, Materials and Methods, Figures, Data Availability Section)
New materials and reagents need to be available; do any restrictions apply?	Not Applicable	

Antibodies	Information included in the manuscript?	In which section is the information available? (Reagents and Tools Table, Materials and Methods, Figures, Data Availability Section)
For **antibodies** provide the following information:<br>- Commercial antibodies: RRID (if possible) or supplier name, catalogue number and or/clone number<br>- Non-commercial: RRID or citation	Not Applicable	

DNA and RNA sequences	Information included in the manuscript?	In which section is the information available? (Reagents and Tools Table, Materials and Methods, Figures, Data Availability Section)
**Short novel DNA or RNA including primers, probes**: provide the sequences.	Yes	in reagenst & tools table (Appendix Table S5)

Cell materials	Information included in the manuscript?	In which section is the information available? (Reagents and Tools Table, Materials and Methods, Figures, Data Availability Section)
**Cell lines:** Provide species information, strain. Provide accession number in repository **OR** supplier name, catalog number, clone number, and/**OR** RRID.	Not Applicable	
**Primary cultures:** Provide species, strain, sex of origin, genetic modification status.	Not Applicable	
Report if the cell lines were recently **authenticated** (e.g., by STR profiling) and tested for mycoplasma contamination.	Not Applicable	

Experimental animals	Information included in the manuscript?	In which section is the information available? (Reagents and Tools Table, Materials and Methods, Figures, Data Availability Section)
**Laboratory animals or Model organisms:** Provide species, strain, sex, age, genetic modification status. Provide accession number in repository **OR** supplier name, catalog number, clone number, **OR** RRID.	Yes	Methods (in reagenst & tools table)
**Animal observed in or captured from the field:** Provide species, sex, and age where possible.	Not Applicable	
Please detail **housing and husbandry conditions**.	Not Applicable	

Plants and microbes	Information included in the manuscript?	In which section is the information available? (Reagents and Tools Table, Materials and Methods, Figures, Data Availability Section)
**Plants:** provide species and strain, ecotype and cultivar where relevant, unique accession number if available, and source (including location for collected wild specimens).	Not Applicable	
**Microbes:** provide species and strain, unique accession number if available, and source.	Yes	in reagenst & tools table

Human research participants	Information included in the manuscript?	In which section is the information available? (Reagents and Tools Table, Materials and Methods, Figures, Data Availability Section)
If collected and within the bounds of privacy constraints report on age, sex and gender or ethnicity for all study participants.	Not Applicable	

Core facilities	Information included in the manuscript?	In which section is the information available? (Reagents and Tools Table, Materials and Methods, Figures, Data Availability Section)
If your work benefited from core facilities, was their service mentioned in the acknowledgments section?	Yes	Acknowledgments

Design

Study protocol	Information included in the manuscript?	In which section is the information available? (Reagents and Tools Table, Materials and Methods, Figures, Data Availability Section)
If study protocol has been **pre-registered, provide DOI in the manuscript**. For clinical trials, provide the trial registration number **OR** cite DOI.	Not Applicable	
Report the **clinical trial registration number** (at ClinicalTrials.gov or equivalent), where applicable.	Not Applicable	

Laboratory protocol	Information included in the manuscript?	In which section is the information available? (Reagents and Tools Table, Materials and Methods, Figures, Data Availability Section)
Provide DOI OR other citation details if **external detailed step-by-step protocols** are available.	Not Applicable	

Experimental study design and statistics	Information included in the manuscript?	In which section is the information available? (Reagents and Tools Table, Materials and Methods, Figures, Data Availability Section)
Include a statement about **sample size** estimate even if no statistical methods were used.	Yes	Statistical analyses
Were any steps taken to minimize the effects of subjective bias when allocating animals/samples to treatment (e.g. **randomization procedure**)? If yes, have they been described?	Not Applicable	
Include a statement about **blinding** even if no blinding was done.	Yes	Statistical analyses
Describe **inclusion/exclusion criteria** if samples or animals were excluded from the analysis. Were the criteria pre-established?<br><br>If sample or data points were omitted from analysis, report if this was due to attrition or intentional exclusion and provide justification.	Not Applicable	
For every figure, are **statistical tests** justified as appropriate? Do the data meet the assumptions of the tests (e.g., normal distribution)? Describe any methods used to assess it. Is there an estimate of variation within each group of data? Is the variance similar between the groups that are being statistically compared?	Yes	in Statistical analyses and in the respective figure legends.

Sample definition and in-laboratory replication	Information included in the manuscript?	In which section is the information available? (Reagents and Tools Table, Materials and Methods, Figures, Data Availability Section)
In the figure legends: state number of times the experiment was **replicated** in laboratory.	Yes	in the respective figure legends.
In the figure legends: define whether data describe **technical or biological replicates**.	Yes	in the respective figure legends.

Ethics

Ethics	Information included in the manuscript?	In which section is the information available? (Reagents and Tools Table, Materials and Methods, Figures, Data Availability Section)
Studies involving **human participants**: State details of **authority granting ethics approval** (IRB or equivalent committee(s), provide reference number for approval.	Not Applicable	
Studies involving **human participants**: Include a statement confirming that **informed consent** was obtained from all subjects and that the experiments conformed to the principles set out in the WMA Declaration of Helsinki and the Department of Health and Human Services Belmont Report.	Not Applicable	
Studies involving **human participants: For** publication of **patient photos**, include a statement confirming that consent to publish was obtained.	Not Applicable	
Studies involving experimental **animals**: State details of **authority granting ethics approval** (IRB or equivalent committee(s), provide reference number for approval. Include a statement of compliance with ethical regulations.	Not Applicable	
Studies involving **specimen and field samples:** State if relevant **permits** obtained, provide details of authority approving study; if none were required, explain why.	Not Applicable	

Dual Use Research of Concern (DURC)	Information included in the manuscript?	In which section is the information available? (Reagents and Tools Table, Materials and Methods, Figures, Data Availability Section)
Could your study fall under dual use research restrictions? Please check biosecurity documents and list of **select agents and toxins** (CDC): https://www.selectagents.gov/sat/list.htm	Not Applicable	
If you used a select agent, is the security level of the lab appropriate and reported in the manuscript?	Not Applicable	
If a study is subject to dual use research of concern regulations, is the name of the **authority granting approval and reference number** for the regulatory approval provided in the manuscript?	Not Applicable	

Reporting

The MDAR framework recommends adoption of discipline-specific guidelines, established and endorsed through community initiatives. Journals have their own policy about requiring specific guidelines and recommendations to complement MDAR.

Adherence to community standards	Information included in the manuscript?	In which section is the information available? (Reagents and Tools Table, Materials and Methods, Figures, Data Availability Section)
State if relevant guidelines or checklists (e.g., **ICMJE, MIBBI, ARRIVE, PRISMA**) have been followed or provided.	Not Applicable	
For **tumor marker prognostic studies,** we recommend that you follow the **REMARK** reporting guidelines (see link list at top right). See author guidelines, under 'Reporting Guidelines'. Please confirm you have followed these guidelines.	Not Applicable	
For **phase II and III randomized controlled trials**, please refer to the **CONSORT** flow diagram (see link list at top right) and submit the CONSORT checklist (see link list at top right) with your submission. See author guidelines, under 'Reporting Guidelines'. Please confirm you have submitted this list.	Not Applicable	

Data Availability

Data availability	Information included in the manuscript?	In which section is the information available? (Reagents and Tools Table, Materials and Methods, Figures, Data Availability Section)
Have **primary datasets** been deposited according to the journal's guidelines (see 'Data Deposition' section) and the respective accession numbers provided in the Data Availability Section?	Yes	Data availability
Were **human clinical and genomic datasets** deposited in a public access-controlled repository in accordance to ethical obligations to the patients and to the applicable consent agreement?	Not Applicable	
Are **computational models** that are central and integral to a study available without restrictions in a machine-readable form? Were the relevant accession numbers or links provided?	Not Applicable	
If publicly available data were reused, provide the respective **data citations in the reference list**.	Not Applicable	
