# Supplementary material for: The calcium-binding protein CSE links Ca2+ signaling with cell-cell communication in cyanobacteria
Source: EMBO J. 2026 Aug 13;45(18):6630–54. doi: 10.1038/s44318-026-00893-y (PMC13578307; doi:10.1038/s44318-026-00893-y)
Supplement: Supplementary file 4 [file 44318_2026_893_MOESM3_ESM.zip › Movie EV1/Legand Movie EV1.docx]

**Expanded View Movie Legend**

**Movie EV1:** Cryo-tomogram of a septal region of wildtype *Nostoc* followed by segmentation, related to (Fig. 6A) and shows normal abundance of septal junctions. Septal junctions are colored in lime green; thylakoid membranes in dark green; peptidoglycan layer and cytoplasmic membranes in gray; granules in yellow. Bar, 100 nm.
