# Supplementary material for: The calcium-binding protein CSE links Ca2+ signaling with cell-cell communication in cyanobacteria
Source: EMBO J. 2026 Aug 13;45(18):6630–54. doi: 10.1038/s44318-026-00893-y (PMC13578307; doi:10.1038/s44318-026-00893-y)
Supplement: Supplementary file 5 [file 44318_2026_893_MOESM4_ESM.zip › Movie EV3/Legand Movie EV3.docx]

**Expanded View Movie Legend**

**Movie EV3.** Cryo-tomogram of a septal region of Δ*cse* mutant followed by segmentation, related to (Fig. EV6), which did not reveal any septal junctions. Thylakoid membranes are colored in dark green; peptidoglycan layer and cytoplasmic membranes in gray; granules in yellow. Bar, 100 nm.
